# Central composite design and mechanism of antibiotic ciprofloxacin photodegradation under visible light by green hydrothermal synthesized cobalt-doped zinc oxide nanoparticles

**DOI:** 10.1038/s41598-024-58961-4

**Published:** 2024-04-21

**Authors:** Mohamed A. Hassaan, Asmaa I. Meky, Howida A. Fetouh, Amel M. Ismail, Ahmed El Nemr

**Affiliations:** 1 Environment Division, National Institute of Oceanography and Fisheries (NIOF), Kayet Bey, Elanfoushy, Alexandria Egypt; 2 Department of Chemistry, Faculty of Science, Alexandria University, Alexandria, Egypt; 3 Alexandria Higher Institute of Engineering and Technology, Alexandria, 21311 Egypt

**Keywords:** Cobalt doped ZnO NPs, Synthesis photocatalysis, Ciprofloxacin, *P. capillacea*, CCD, RSM, Environmental chemistry, Chemical engineering, Catalyst synthesis

## Abstract

In this research, different Co^2+^ doped ZnO nanoparticles (NPs) were hydrothermally synthesized by an environmentally friendly, sustainable technique using the extract of *P. capillacea* for the first time. Co-ZnO was characterized and confirmed by FTIR, XPS, XRD, BET, EDX, SEM, TEM, DRS UV–Vis spectroscopy, and TGA analyses. Dislocation density, micro strains, lattice parameters and volume of the unit cell were measured using XRD results. XRD suggests that the average size of these NPs was between 44.49 and 65.69 nm with a hexagonal wurtzite structure. Tauc plot displayed that the optical energy bandgap of ZnO NPs (3.18) slowly declines with Co doping (2.96 eV). Near complete removal of the ciprofloxacin (CIPF) antibiotic was attained using Green 5% of Hy-Co-ZnO in the existence of visible LED light which exhibited maximum degradation efficiency (99%) within 120 min for 30 ppm CIPF initial concentration. The photodegradation mechanism of CIPF using Green Hy-Co-ZnO NPs followed the Pseudo-first-order kinetics. The Green Hy-Co-ZnO NPs improved photocatalytic performance toward CIPF for 3 cycles. The experiments were designed using the RSM (CCD) method for selected parameters such as catalyst dosage, antibiotic dosage, shaking speed, and pH. The maximal CIPF degradation efficiency (96.4%) was achieved under optimum conditions of 39.45 ppm CIPF dosage, 60.56 mg catalyst dosage, 177.33 rpm shaking speed and pH 7.57.

## Introduction

Pharmaceuticals are being used more frequently to cure illnesses in humans and other species because of the world population's ongoing growth and the resulting rise in demand. High levels of contamination in the ecosystem are a result of the increased use of pharmaceuticals in daily life and their release from wastewater of hospitals and effluents of the pharmaceutical industry^1–3^. Ciprofloxacin (CIPF), is an antibiotic medicine from the fluoroquinolone family that is often administered all over the world^3,4^. Eliminating CIPF from contaminated water has become crucial since it is believed to be causing several long- and short-term problems, including serious harm to ecosystems and human health, even at low levels of contamination^5–7^. The removal and degradation of antibiotics from groundwater and surface water have been accomplished using a variety of advanced oxidation processes (AOPs). These AOPs include the UV/H_2_O_2_ process, electrochemical approach, electro-Fenton process, photo-Fenton process, and photocatalysis. Heterogeneous photocatalysis has been exploited for the mineralization and degradation of a wide variety of organic contaminants in these processes^8–10^. Numerous catalysts, including TiO_2_, ZnO, ZnS, Al_2_O_3_, Fe_2_O_3_, and CdS, have been employed for this purpose^8^. ZnO material, one of the II-VI compound semiconductors, has received the most attention due to its wide band gap (3.2 eV) and increased absorption near the UV range for a variety of applications, including dye-sensitive sensors, solar cells (DSSCs), photocatalysis, light-emitting diodes (LEDs), and antibacterial^11,12^. Researchers have lately become interested in these applications because ZnO materials have the photocatalytic capacity to break down organic pollutants, convert carbon dioxide to fuel, and create hydrogen^11^.

Cobalt is regarded as a useful metal for doping the ZnO matrix because of its rich electron status and high solubility^13,14^. Co^2+^ ions can easily replace Zn^2+^ ions in the ZnO lattice because cobalt's ionic radius is comparable to that of zinc^15^. The separation of photo-generated charges is improved by the non-full d orbitals of the Co^2+^ ions, which serve as electron traps in the ZnO matrix^16^. Cobalt dopant inclusion causes the band gap to create new energy levels that can be stimulated by visible light^16^. This has to do with the interaction between localized d electrons of transition metal ions and ZnO band edge electrons known as the sp-d spin exchange interaction^17^.

Important medical discoveries in the history of humanity have been credited to applications of nanomaterials in the biomedical field^18,19^. Metallic nanoparticles differ from their bulk counterparts in terms of their physical and chemical characteristics because of their high surface ratio^20^. Nowadays, academics and industry researchers favor the biocompatible, economical, and secure method of producing nanoparticles (NPs) from biomass, such as fungi, plant extracts, algae, and bacteria, employing green technologies^21–24^. Magnetic nanoparticles are frequently produced utilizing conventional techniques such as microwave-assisted synthesis, chemical processes, hard template guiding techniques, sol–gel, and thermal methods due to their wide dispersion in several applications.

When using the chemical synthesis approach, harmful chemical byproducts or extreme heat and pressure are produced^25–27^. However, common experimental methods that involve the use of high reactive reducing, organic solvents, and capping agents have the potential to have harmful effects on the environment^28^. As a result, the green synthesis of NPs has been suggested as an environmentally benign substitute for chemical and physical processes employing microbes, alga, enzymes, and plants^29^. Researchers suggested several potential plant extracts and fungal biomasses that could be applied in the green preparation of ZnO NPs, such as the Neem leaf extract^30^, black dried lemon peel aqueous extract^31^, B. tinctoria leaves and fruit extract^25^ and *Moringa oleifera* leaf extract by hydrothermal method^32^. *P. capillacea* is a typical marine biomaterial found in the Mediterranean, where a sizable amount is annually produced on rocks by the coast and in shallow water. El Nemr et al.^33^ stated that *P. capillacea* has exceptional metal adsorption properties and is a typical marine biomaterial in the region. *P. capillacea* is a marine red alga in which chlorophyll is hidden by the pigment phycoerythrin. They are always multicellular, vary in size from small to medium, and have hollow fronds with a cartilaginous texture^34^. So, it was applied in the green formation of ZnO NPS in this work.

Response surface methodology (RSM) has become widely employed in recent years to examine how various independent variables affect the response variables in chemical processes. Many experimental designs have been used to estimate the process, including Box-Behnken design (BBD), Doehlert matrix (DM), and central composite design (CCD)^35,36^. The best model among these is CCD since it uses statistics to its advantage to establish the ideal circumstances and influence of each independent variable^37^. Therefore, the RSM-CCD model was used to shorten process iterations, minimize costs, and save time. This work successfully prepared a novel Green hydrothermal Co-doped ZnO NPs with different concentrations of cobalt (5–15%) using *P. capillacea* water extract. Moreover, the CIPF efficient removal from water by photodegradation processes in the presence of visible light irradiation was optimized via the RSM-CCD model. In the authors’ knowledge, this is the first work that uses RSM-CCD for optimizing photocatalytic degradation of CIPF using Co-doped ZnO which was hydrothermally synthesized in green friendly, sustainable way using the extract of *P. capillacea* as green reducing agent and capping agent.

## Materials and methods

### Chemicals and equipment

Zinc acetate dihydrate, cobalt acetate tetrahydrate, NaOH, isopropanol (IPA), Na-EDTA and Benzoquinone (BQ) were purchased from Sigma Aldrich, USA. CIPF was purchased as CIPF 200 mg/100 mL I.V. infusion solution from Amirya Pharmaceuticals, Egypt. Fresh red algal biomass of *P. capillacea* species was collected from the Mediterranean coast, Alexandria, Egypt. All chemicals used are of analytical grade and used as received without further purification. The following instruments were applied to identify the Green Hy-ZnO NPs and Green Hy-Co-ZnO NPs photocatalysts. Green Hy-ZnO NPs and Green Hy-Co-ZnO NPs crystallinity, and average crystal size were confirmed by Bruker Meas Srv (D2-diffractometer that controls at 30 kV, 10 mA using Cu tube λ = 1.5418 Å and 2*θ* with a temperature range of 5° to 80°) were used. Fourier transform infrared (FTIR) spectroscopy model VERTEX70 linked to platinum ATR model V-100, Bruker, Germany, in the 400–4000 cm^−1^ wavenumber range. SEM (SEM-JEOL, IT 200, Japan) equipped with Energy dispersive X-ray spectroscopy (EDX) elemental analysis, was used to determine the materials' morphology and surface characteristics. TEM (JTM 1400 plus, Japan) was used to determine the size and shape of the nanostructures. UV–Visible, GBC Cintra 3030 at the range 190–900 nm spectrophotometer was used to measure the optical absorbance of these samples. Using the BELSORP—Mini II from BEL Japan, Inc., the mean pore diameter and specific surface area (BET, Brunauer Emmett-Teller) were measured. Thermal studies of the samples were performed using the SDT650-Simultaneous Thermal analyzer apparatus at a ramping temperature of 10 °C per minute. Monochromatic X-ray Al *K*-alpha radiation with a spot size of 400 µm, a pressure of 10^–9^ mbar, a full spectrum pass energy of 200 eV, and a narrow spectrum energy of 50 eV was used to gather XPS data on *K*-alpha (Thermo Fisher Scientific, USA).

### Preparation of *P. capillacea* extract

*P. capillacea* fresh red algal biomass was collected, cleansed with tap and saltwater, and then washed with distilled water. The cleaned algal biomass was then dried for 72 h at 105 °C after being sun-dried for two days, and the dried red algae was ground for use. 250 ml of distilled water (DW) was combined with 10 g of the resultant powder. On a hot plate, the mixture was agitated at 75 °C for two hours before being filtered through filter paper. To prepare the *P. capillacea* extract for processing, it was kept at 20 °C^33^.

### ***Synthesis of ZnO and Co-doped ZnO (Zn***_***1−***x_***Co***_x_***O)***

Zn (CH_3_COO)_2_·2H_2_O (0.5 M) (solution-A) was added to 90 mL of DW and 10 mL of *P. capillacea* extract under continuous stirring at 60 °C for 30 min followed by the addition of 0.5 M NaOH dropwise until a white precipitate color was showed. The solution was vigorous stirring for 60 min at the same temperature. Before being transferred to a Teflon-lined autoclave, the solution mixture was kept for 30 min for ultrasonication treatment in an ultrasonic water bath. In an electric oven, the hydrothermal syntheses took place over 12 h at 150 °C. In the next steps, the obtained white precipitate was separated, filtered, washed several times with distilled water and EtOH, dried, and finally calcined at 500 °C^14^. Green Hy-ZnO NPs' pale white powder was painstakingly gathered and stored till use. The Green Hy-Co-ZnO (Zn_1-*x*_Co_*x*_O) NPs were synthesized via the hydrothermal method in the same way. Different ratios (5, 10, and 15% of Co/ZnO) were synthesized by dissolving the desired amount of Co (CH_3_COO)_2_·4H_2_O (solution-B) in distilled water and *P. capillacea* extract under stirring for 30 min. Solution-B was then added dropwise to Solution-A followed by applying the same procedures as for the synthesis of Green Hy-ZnO NPs.

### Photocatalytic activity

To determine the best catalyst performance, a specific amount of 100 mg of Green Hy-ZnO NPs, 5, 10, and 15% of Green Hy-Co-ZnO NPs was added to a Pyrex glass beaker containing 100 mL of CIPF with concentrations of 30 ppm at neutral pH for 2 h under (150 W LED light lamp) as the visible light source in a shaker incubator at room temperature and the removal efficiency was measured. In a typical photocatalytic degradation experiment, the CIPF solution was placed in a 100 mL flask beaker along with the photocatalyst, and the combination was left in the dark for 30 min to establish the adsorption–desorption equilibrium. Then, visible light was shone on the reaction medium containing the photocatalyst and CIPF antibiotic solution. By taking 2 mL of the aliquot CIPF antibiotic solution at regular time intervals. A UV–Vis spectrophotometer (model Pg/T80 UV/Vis) was used for CIPF concentration analysis at a *λ*_max_ 270 nm after it had been centrifuged at 6000 rpm for 30 min.

1 $$Degradation efficiency=\frac{{C}_{0}-{C}_{t}}{{C}_{0}}\times 100$$

**Figure 1:**
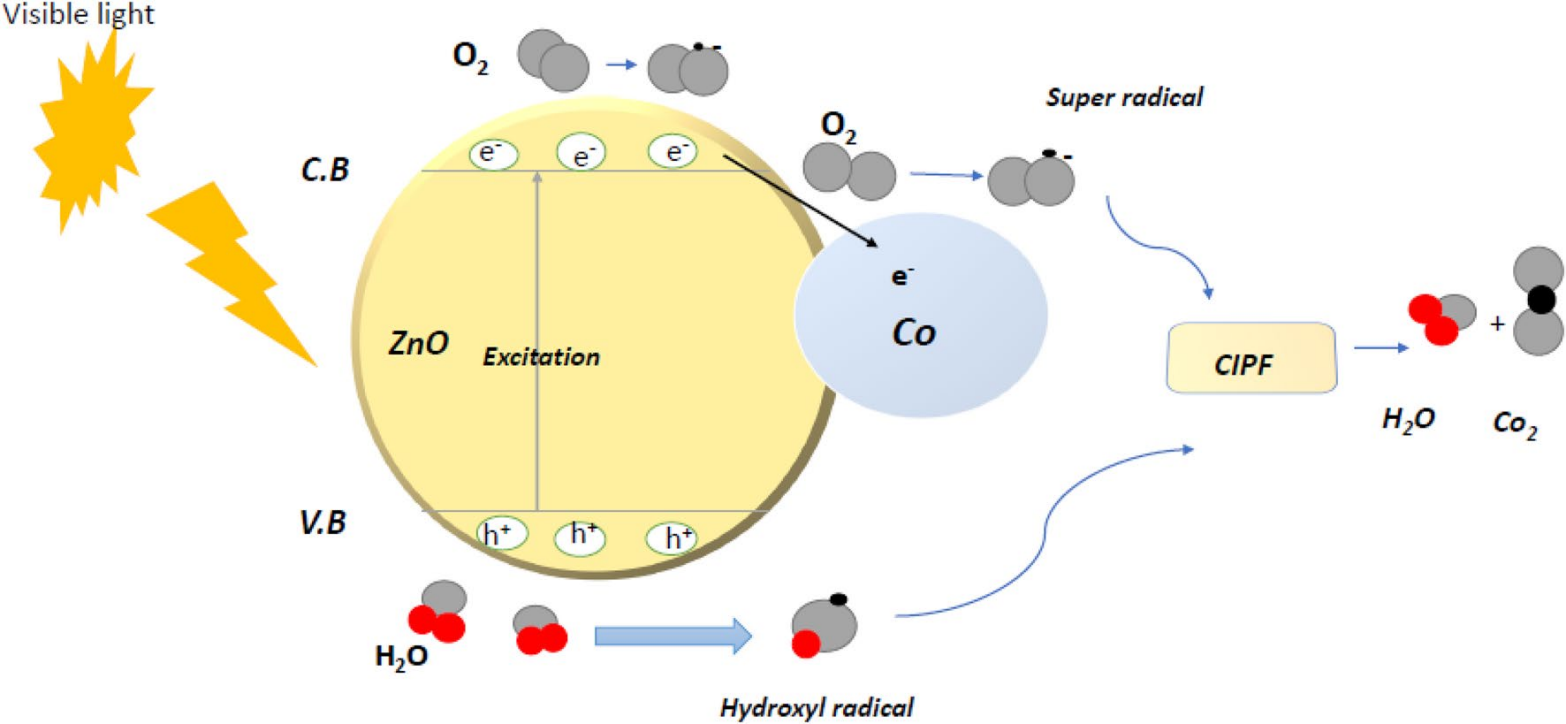
Mechanism of photocatalytic activity of Green Hy-Co-ZnO NPs.

*C*_0_ stands for the CIPF initial concentration, and *C*_t_ refers to the final concentration of CIPF antibiotic in solution at the specified time intervals after irradiation. To determine the ideal circumstances for effective photocatalytic degradation, the photodegradation parameters of pH, catalyst doses, antibiotic concentrations, temperature, and shaking speed were optimized. Figure 1 presents a summary of the potential photogenerated charge transfer events that might occur in the Green Hy-Co-ZnO NPs photocatalysts during the breakdown of the antibiotic.

Photodegradation of ciprofloxacin was tested in visible light without any catalyst and photodegradation of ciprofloxacin with catalyst was tested in the dark and both showed no degradation activity.

### Radical scavenger

Three scavengers (10 mM Na-EDTA, 1 mM IPA, and 1 mM BQ, individually) were added to a 100 mL, 30 ppm CIPF solution to quench the photo-generated species (holes (h^+^), hydroxyl radicals (**·**OH), and superoxide radicals (**·**O_2_^–^ ), which are each responsible for catalytic degradation, respectively^38^.

### Experimental design

To investigate the impact of the effective components and recognize the optimal conditions for the photocatalytic degradation effectiveness of the antibiotic ciprofloxacin using Green Hy-Co-ZnO NPs, the RSM design-CCD design was developed using Design Expert Software (13.0.5.0). RSM studied how effective operating variables interacted and related to each other to determine the optimal conditions for the photocatalytic degradation of the CIPF antibiotic.^39^. In this study, the impacts of four variables and five levels, including pH (D), shaking duration (C), CIPF antibiotic dose (B), and photocatalyst dosage (g/L) (A), were detected. The antibiotic's effectiveness at degrading (*R*%) after 120 min is the observed response.

RSM analysis recommended 30 experiments while taking into account all possible combinations of the input variables, of which 6 replications to a central point were included to ensure proper data optimization. The interactions between the independent and dependent components were then examined using a quadratic model^39^. The experimental intervals and various degrees of experimental designs for antibiotic removal that are taken into consideration in this investigation are shown in Table 1.

**Table 1:** Values at the low and high levels for the independent variables.

Factor	Name	Units	Minimum	Maximum	Mean	Std. Dev. +
A	Catalyst dosage	mg	20	100	60.0	18.19
B	Antibiotic dosage	mg/L	10	50	30.0	9.10
C	Shaking speed	rpm	50	250	150.0	45.49
D	pH		3	11	7.0	1.82

## Results and discussion

### FTIR analysis

**Figure 2:**
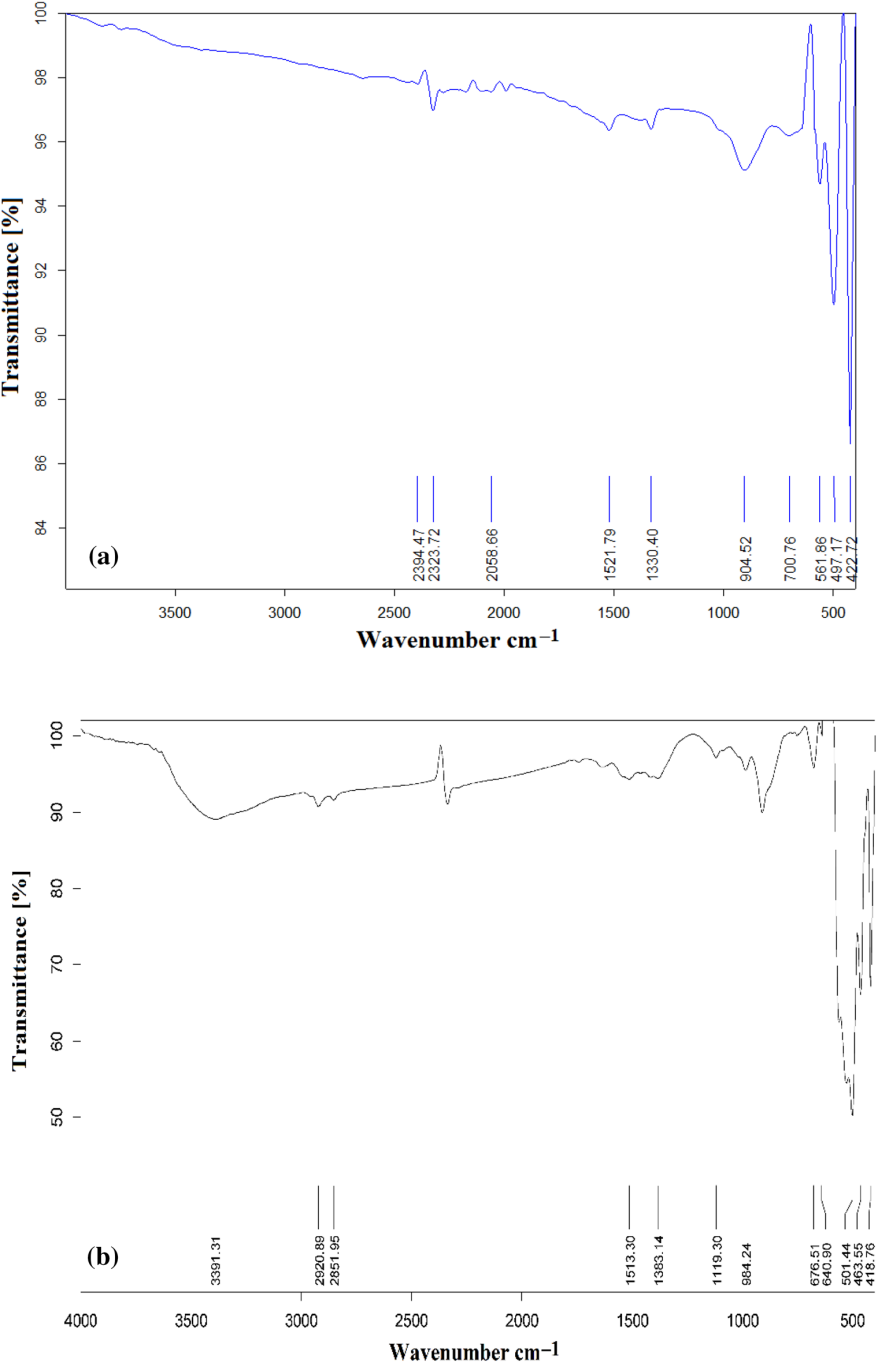
FTIR spectra of (**a**) Green Hy-ZnO NPs, (**b**) Green 5% Hy-Co-ZnO NPs catalyst.

The FTIR spectra of Green Hy-ZnO NPs and Green 5% Hy-Co-ZnO NPs in the wavenumber range of 400–4000 cm^–1^ is represented in Fig. 2. The peaks at 422, 497, and 561 cm^–1^ for Green Hy-ZnO NPs can be recognized as Zn–O stretching, whereas the band at 2394 cm^–1^ can be attributed to atmospheric CO_2_. C=O stretching is responsible for the absorbance peak at 1521 cm^–1^^40,41^. The broadband at 1330 cm^–1^ was ascribed to the deformation vibrations of C-H^31^. The band noticed at 904 cm^–1^ corresponds to the C–OH group^42^. Strong FTIR bands at 418, 463, and 501 cm^–1^ for Green 5% Hy-Co-ZnO NPs are attributed to Zn–O tetrahedron stretching vibrations^43–45^. The Zn–O bond lengths slightly alter as Co^2+^ ions take the place of Zn^2+^ ions in the ZnO host, causing a change in wavenumber. The stretching vibration of the hydrogen bond (O–H) band from the surface of the NPs causes a strong and wide absorption band to form at about 3391 cm^–1^^46^. Hybrid asymmetric and symmetric C = O stretching resonance is present in the band at 1423 cm^–1^. C–H bending from various NPs' surface dangling bonds is represented by the FTIR bands 640, 676, 901, and 984 cm^–1^^47–50^. Additional peaks at 2851–2920 cm^−1^ are due to the CH_2_ (C–H) groups in acetate vibrating^42^. As previously mentioned, 5% Co^2+^ ions doping NPs have remarkably similar FTIR spectra. The insertion of Co^2+^ into the ZnO lattice, however, seems to have caused a little shift.

### Surface area analysis

For determination of surface area (SA), the N_2_ adsorption–desorption isotherm for ZnO and Green 5% Hy-Co-ZnO NPs are shown in Fig. S1 the four materials have type IV isotherm (IUPAC classification)^51^. Additionally, the existence of mesopores in the structure of ZnO and Co-ZnO nanoparticles is shown by their extremely small hysteresis loops under moderate relative pressure^52,53^. Notably, the maximum volumes absorbed for Green Hy-ZnO, Green 5, 10, and 15% Hy-Co-ZnO NPs are 1.5586, 1.1447, 1.2203, and 1.6672 cm^3^/g, respectively. The specific SA calculated from the adsorption–desorption data obtained from the isotherms was 6.7837, 4.9823, 5.3115, and 7.2564 m^2^/g, respectively. The determination of the pore distribution and pore size of Green Hy-ZnO, Green 5, 10, and 15% Hy-Co-ZnO NPs have been calculated by Barret-Joyner-Halenda (BJH) pore size distribution which are depicted in Table 2 and Table S1.

**Table 2:** Analysis of the surface area of Green Hy-ZnO, Green 5% Hy-Co-ZnO NPs.

		Green Hy-ZnO NPs	Green 5% Hy-Co-ZnO NPs
BET	*a*_s, BET_ (m^2^∕g)	6.7837	4.9823
	*V*_m_ (cm^3^ STP)/g)	1.5586	1.1447
	Mean pore diameter (*P*_m_) (nm)	10.877	11.67
	Volume of total pore (*V*_T_) (cm^3^/g)	0.018446	0.014544
BJH	*V*_p_ (cm^3^/g)	0.018437	0.014868
	*a*_p_ (m^2^/g)	6.8287	5.5432

### SEM analysis

**Figure 3:**
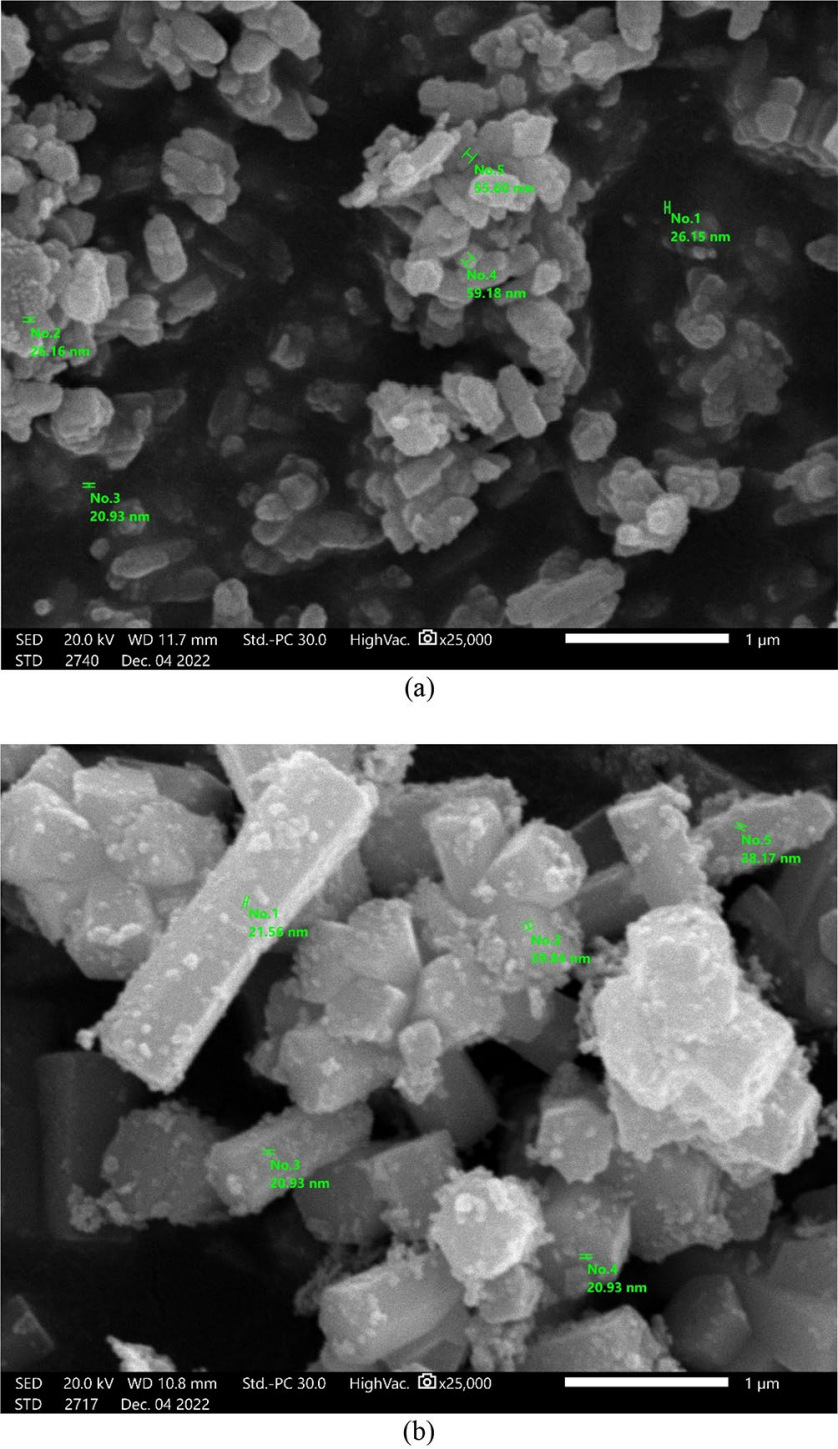
SEM analysis of (**a**) Green Hy-ZnO NPs, (**b**) Green 5% Hy-Co-ZnO NPs catalyst.

The SEM morphologies of Green Hy-ZnO and Green 5% Hy-Co-ZnO nanoparticles are shown in Fig. 3. Figure 3a depicts an image of an almost nanoparticle with a uniform size distribution of ZnO nanocrystals. Green Hy-ZnO nanoparticles are shown as nanosheets. Figure 3b shows Green 5% Hy-Co-ZnO nanoparticles as cylindrical. ZnO makes up the agglomeration when nanoparticles of Co are scattered across its surface. The high degree of agglomeration in the nanoparticles ought to help boost the contact surface for increased reactant diffusivity, which is especially favorable for catalytic activity. As presented in Fig. 3, SEM micrographs also expose that Co-ZnO 5% NPs decrease in average particle size.

### EDX analysis

**Figure 4:**
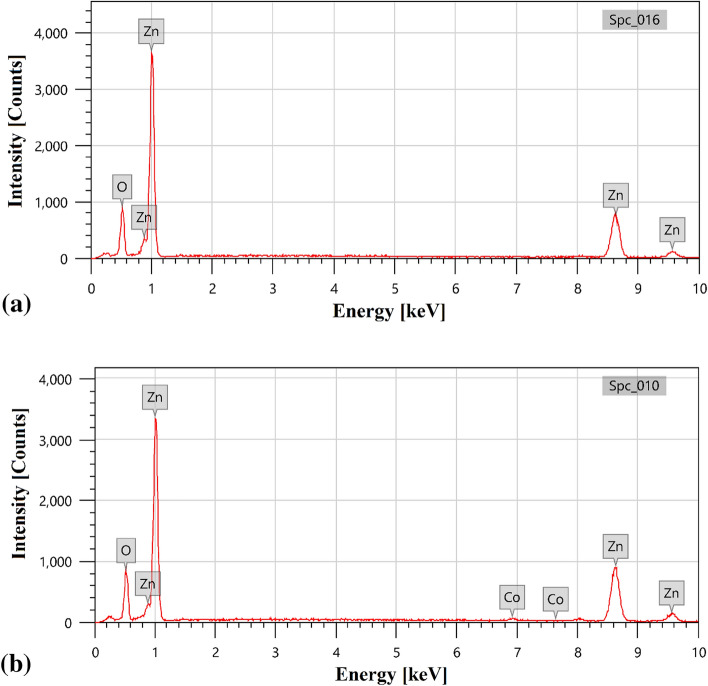
EDX spectrum of (**a**) Green Hy-ZnO NPs, (**b**) Green 5% Hy-Co-ZnO NPs catalyst.

The fundamental evaluation of Green Hy-ZnO and Green 5% Hy-Co-ZnO nanoparticles was examined in the current study using EDX analysis. The result is displayed in Fig. 4a. It expresses the EDX pattern of Green Hy-ZnO NPs confirming the existence of ZnO elements with strong signal at the Zn region and suggesting the presence of Zn and O_2_ with the atomic weights of 49.550 ± 60 and 50.450 ± 67, respectively. Green 5% Hy-CO-ZnO sample has Zn, Co, and O with atomic weights of 53.200 ± 59, 0.790 ± 06, and 46.010 ± 61, respectively, according to the EDX spectra (Fig. 4b). High-purity samples are indicated by the absence of additional elements in the spectra (Table 3).

**Table 3:** Element analysis of Green Hy-ZnO NPs, Green 5% Hy-Co-ZnO NPs catalyst.

Element	Green Hy-ZnO NPs		Green 5% Hy-Co-ZnO NPs	
	Mass%	Atom%	Mass%	Atom%
Zn	80.05 ± 0.97	49.55 ± 0.60	81.63 ± 0.91	53.20 ± 0.59
O	19.95 ± 0.27	50.45 ± 0.67	17.28 ± 0.23	46.01 ± 0.61
Co	0.00	0.00	1.09 ± 0.09	0.79 ± 0.06
total	100	100	100	100

### TEM analysis

**Figure 5:**
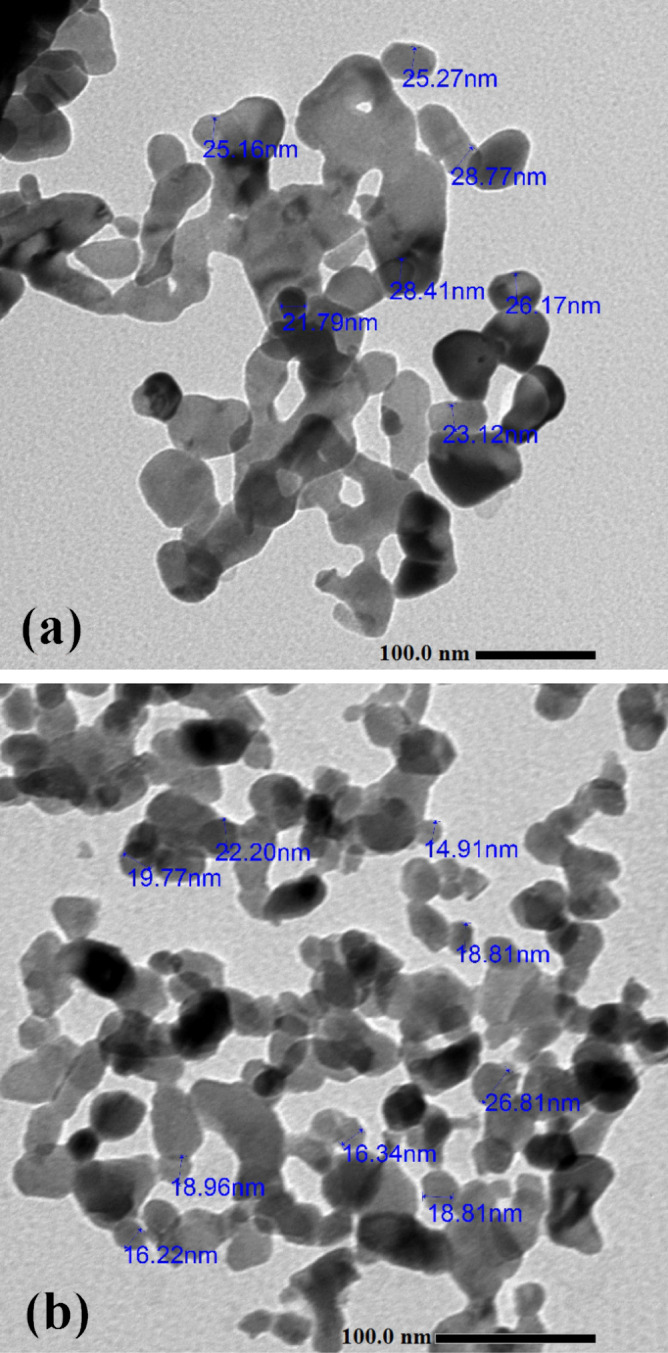
TEM analysis of (**a**) Green Hy-ZnO NPs, (**b**) Green 5% Hy-Co-ZnO NPs catalyst.

Transmission electron micrographs (TEM) were applied to analyze the morphological changes that co-doping caused in the particles. In Fig. 5, representative TEM images of pure ZnO and nano-crystalline samples containing 5% Co are displayed. In Fig. 5a, it is shown that the average size of ZnO nanoparticles was about 30 nm. The Co-doped ZnO nanoparticles in Fig. 5b, on the other hand, had an average size that was about 18 nm smaller than that of pure ZnO. The difference can be attributed to a thermodynamic barrier that the dopant (Co^2+^) creates that slows down ZnO nucleation and prevents it from spreading outward around a doped nano-crystal^54^. Due to the nano-metric crystals' high surface energy, the nanoparticle aggregates are believed to have formed.

### X-ray diffraction (XRD) analysis

**Figure 6:**
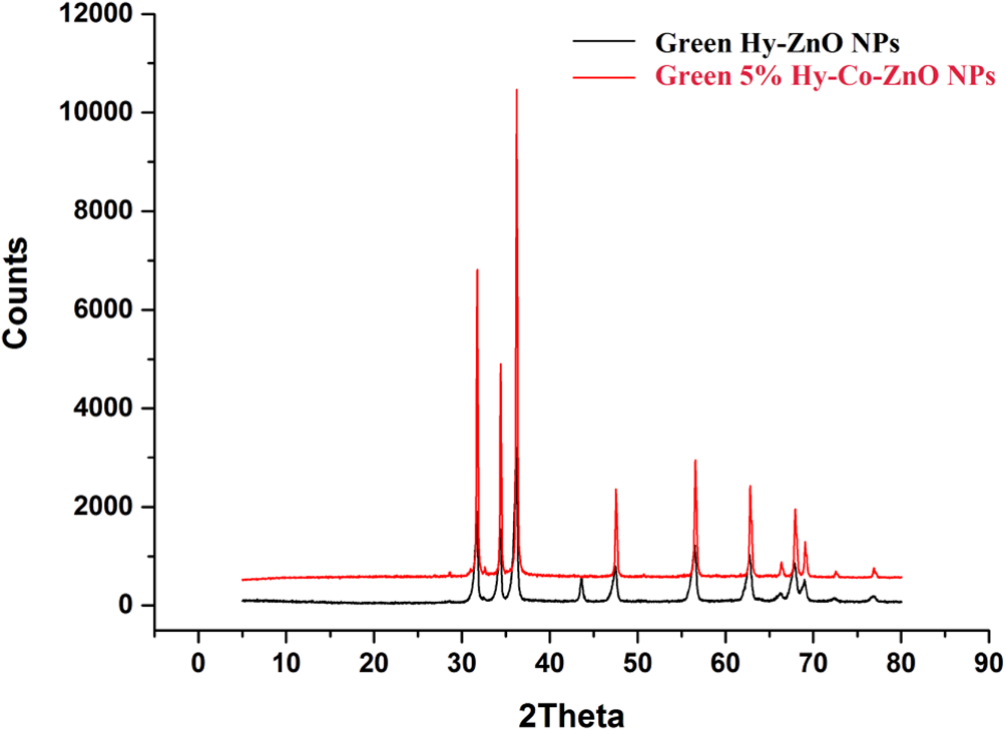
XRD pattern of Green 5% Hy-Co-ZnO NPs and Green Hy-ZnO NPs.

Figure S2 displays the XRD analysis of Green Hy-ZnO NPs and Green Hy-Co-ZnO NPs samples at different concentrations (5, 10, and 15%). The hexagonal quartzite structure of Green Hy-Co-ZnO NPs is established by the strong bands seen in the XRD patterns equivalent to planes (100), (002), (101), (102), (110), (103), (200), (112), (201), (004), and (202). A larger version of the (101) peak's picture, which contrasts with Green Hy-ZnO NPs, can be seen in Fig. 6 demonstrating a minor shift in the location of the peaks. The inclusion of Co^2+^ ions into the ZnO lattice is shown by this change in peak position^55^. The samples are very pure, and it appears that Co-ions are successfully occupied in the lattice site rather than the interstitial site based on the lack of structural alterations and further phase shifts caused by Co doping in ZnO NPs as compared to Green Hy-ZnO NPs^55^.

Using Debye–Scherrer's Eq. (2), the average crystallite size 'D' of the Green Hy-ZnO NPs and Green Hy-Co-ZnO NPs samples (Table S2) has been calculated from the strongest diffraction peak (101)^56^.

2 $$D=\frac{K\uplambda }{\beta COS\theta }$$

where *K* is a constant (*K* = 0.94 for spherical shape), *λ* is the wavelength (1.5417 Å) of X-rays, *β* is the full-width at half-maxima (FWHM) of the diffraction band (in radians) and *θ* is the Bragg’s diffraction angle. Other Different relations used for measurement of properties using XRD results were done according to the following calculation^56^:

The number of defect states in the sample, or dislocation density ‘δ’, may be estimated from crystallite size ‘D’ using the formula (Eq. 3).

3 $$\delta =\frac{1}{{D}^{2}}$$

The following equation (Eq. 4) can be used to compute the microstrain.

4 $$\varepsilon =\frac{ \beta cos\theta }{4}$$

Here, *β* is Full width at half maximum (FWHM), is Bragg's angle, and is the microstrain.

Equation (5) is used to compute the lattice parameters for all hexagonal wurtzite structural samples.

5 $$\frac{1}{d2}=\frac{4}{3} \left(\frac{{h}^{2}+hk+{k}^{2}}{{a}^{2}}\right)+\frac{{l}^{2}}{{c}^{2}}$$

where *h*, *k*, and *l* are Miller indices, a, and c are lattice parameters, d is interplanar spacing, and h, k, and l are Miller indices. The lattice constant 'a' is determined by using relation (1) for the plane (100), lattice constant '*c*' is derived for the plane (004) by using relation (2), and so on. Taking into account the first-order approximation (*n* = 1) for the plane (100). The following Eqs. (6–8) were used to get the volume of the unit cell:

6 $$a=\frac{\lambda }{\sqrt{3 }sin\theta }$$

7 $$c=\frac{\lambda }{sin\theta }$$

8 $$V=\frac{\sqrt{3 } {a}^{2}c}{2}$$

The aforementioned context shows that the average crystalline size grew as cobalt concentration increased. This may be caused by a rise in the Co concentration at the ZnO matrix, which would boost particle nucleation and encourage the creation of Co-doped ZnO NPs grains. This research supports previous findings by other scientists as^11,13,56^, where they noticed that the crystal size of the ZnO increased as cobalt was doped into the material. Table S3 displays the relationship between lattice parameters *a*, and *c* and Co molar concentration. With an increase in Co concentration, relatively little change in ZnO's lattice characteristics is seen. This outcome is related to the systematic replacement of Zn^2+^ ions with Co^2+^ ions without affecting the ZnO crystal structure^56^. This might be because the ionic radii of Zn^2+^ and Co^2+^ ions are almost identical. The study of the lattice strain shift (*ε*) brought on by the addition of Co to ZnO. Most of the time, the increase or decrease in lattice strain with dopant concentration is correlated with the increase or decrease in dislocation density^56^. In the current work, the increase in dislocation density is correlated with the rise in lattice strain on doping (Table S3). As demonstrated in Fig. 6 and Fig. S2, a little shift is also seen in the peaks for the cobalt-doped ZnO samples as a result of the cobalt Co^2+^ being replaced by zinc (Zn^2+^), which is noticeable as a drop in peak (101) intensity. Particle size or lattice strain may be affected by evidence of peak reduction in terms of peak intensity. For Co-ZnO 5% NPs, Table S3 displays the fluctuation in average crystallite size and strain for cobalt content. Increases in lattice parameters (*a*,*c*) and volume are the foundation for the deformation variance shown in Table S3. The mismatch in cobalt and zinc oxide's radii is responsible for the smaller fluctuation that was found. A change in the oxygen parameter, which rises with an increase in cobalt, was caused by the increase in volume (Table S3). The lattice properties of ZnO may alter as a result of these changes in defect density.

### UV–vis and DRS analysis

**Figure 7:**
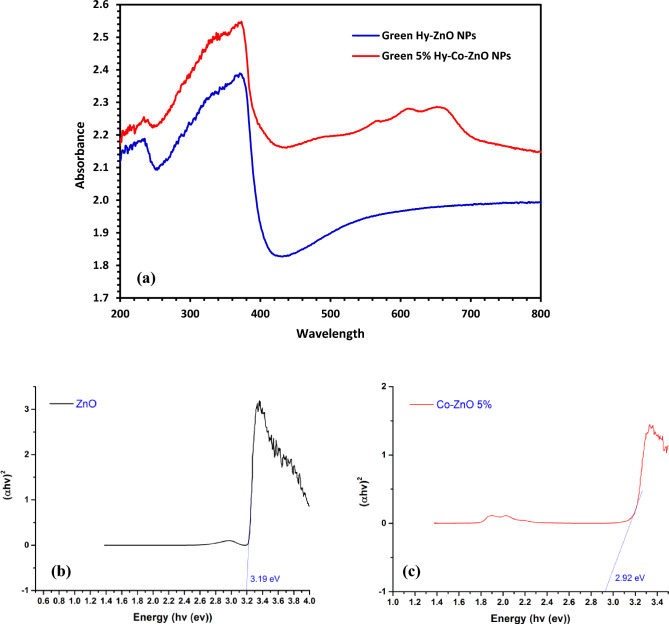
(**a**) UV–DRS spectra of Green Hy-ZnO NPs and Green 5% Hy-Co-ZnO NPs, (**b**) Tauc plots of Green Hy-ZnO NPs and Green 5% Hy-Co-ZnO NPs.

It is feasible to define the energy band gap of Green Hy-ZnO NPs using DRS measurements. Figure 7a displays the DRS spectra of Green 5% Hy-Co-ZnO NPs and Green Hy-ZnO NPs. Without any additional reflection peaks, ZnO is visible with a sharp reflection edge at 374 nm. The Green 5% Hy-Co-ZnO NPs have some extra reflectance peaks in the visible region, and these are typical bands of the Co ion replaced in the lattice of ZnO at 571 nm (2.171 eV), 612 nm (2.026 eV), and 657 nm (1.887 eV), which related to d-d transitions of Co^2+^ ions. These absorptions result from d-d transitions of high spin Co^2+^ ions^19^, which could be assigned to transition from ^4^A2 → ^2^E (G), ^4^A2 → ^4^T1(P), and ^4^A2 → ^2^A1(G), respectively^57^. Using Tauc's relation, the energy bandgap (*Eg*) was determined as follows^57^.

9 $$ \alpha h\vartheta = A\left( {h\vartheta - Eg} \right)^{n} $$

Here, "*A*" is a constant, "*α*" is the absorption coefficient, and "*n*" is a constant that is equal to 2 for an indirect transition and 1/2 for a direct transition. Figure 7b,c shows the (*αhυ*)^2^ values concerning to photon energy (*hυ*). Zn1-xCoxO NPs with x = 0.00 and 5% of Co were discovered to have optical direct energy bandgaps (*Eg*) of 3.18 and 2.96 eV, respectively. The redshift in absorption spectra, which was found, revealed that the *Eg* value decreased as the Co concentration increased. It has been shown that the Co level causes a similar decrease in the bandgap energy^57^.

### X-ray photoelectron spectroscopy (XPS)

**Figure 8:**
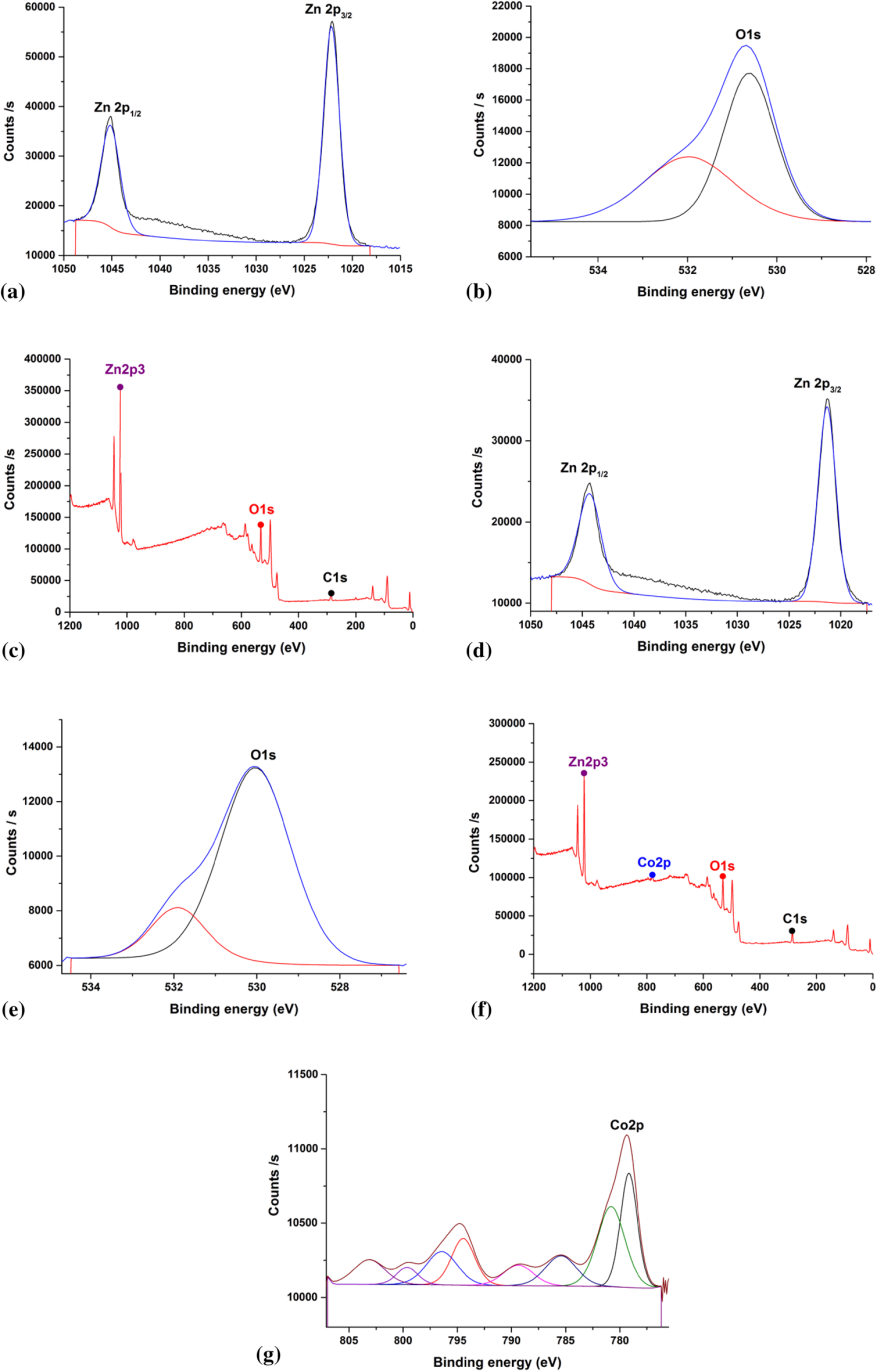
X-ray photoelectron spectra of (**a**–**c**) Green Hy-ZnO NPs, (**d**–**g**) Green 5% Hy-Co-ZnO NPs catalyst.

Utilizing XPS, it was discovered what chemical states certain components had in Green 5% Hy-Co-ZnO NPs and Green Hy-ZnO NPs. The sample's peaks with different binding energies could all be assigned to O, Zn, Co, and C, and no other impurities were discovered, conferring by the XPS survey scan spectra in Fig. 8. The C1s signal is a naturally occurring carbon contamination brought on by the sample's exposure to air before its XPS measurement, which cannot be avoided. The Zn2p spectra in Fig. 8, reveal two peaks with the designations Zn 2p_1/2_ and Zn 2p_3/2_. The roughly 22.9 eV binding energy gap between these peaks supports the Zn^2+^ chemical states^54^. The O1s spectrum was scanned in Fig. 8, which indicated two sub-peaks near the peaks 530 and 531 eV, respectively. The signal at 530 eV was ascribed to O at the oxide lattice, while the signal at 531 eV was given to zinc (OH)^58^. Similar results are shown for the Co 2p XPS wide scan in Fig. 8, with the Co 2p_3/2_ signal centring at 779.16 eV and the Co 2p_1/2_ signal at 780.8 eV. The difference in binding energies between the Co 2p peaks points to the presence of the Co^2+^ ion. Therefore, the XPS analysis exposes that Co^2+^ was doped in the lattice of ZnO by replacing Zn^2+^ with no additional impurities^54^.

### Thermogravimetric analysis

**Figure 9:**
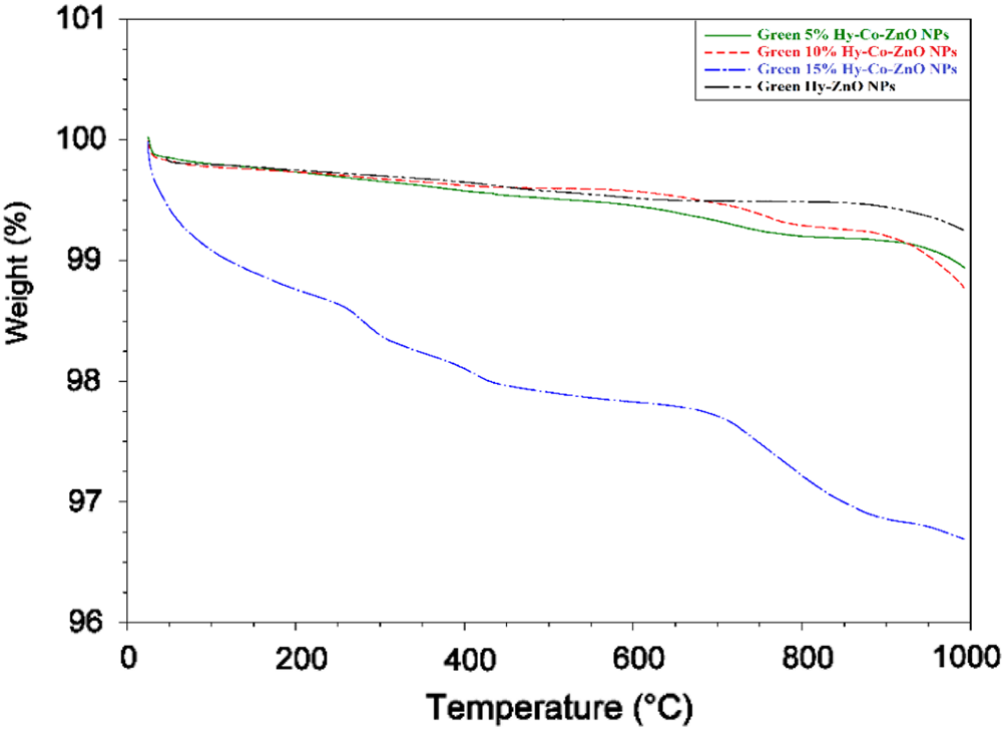
TGA analysis of Green Hy-ZnO NPs and 5, 10, and 15% Hy-Co-ZnO NPs.

A well-known method for detecting the physical, chemical, or breakdown reactions of manufactured materials is thermogravimetric analysis (TGA). The results of testing the thermal stabilities of Green Hy-ZnO NPs, 5, 10, and 15% Hy-Co-ZnO NPs as a function of temperature are exposed in Fig. 9, and further information is available in Fig. S3. As a result, the samples' TGA curve shows evidence of several degradation parts. The first part is related to the water removal from the surface of the samples (50–200 °C), and after 200 °C, the weight loss was slight and was mainly due to moisture degradation and CO_2_ absorption^59^. The weight loss seems to have stabilized, which suggests that temperature has nearly completely removed the contaminants.

## Photocatalytic studies

### Photocatalytic test

**Figure 10:**
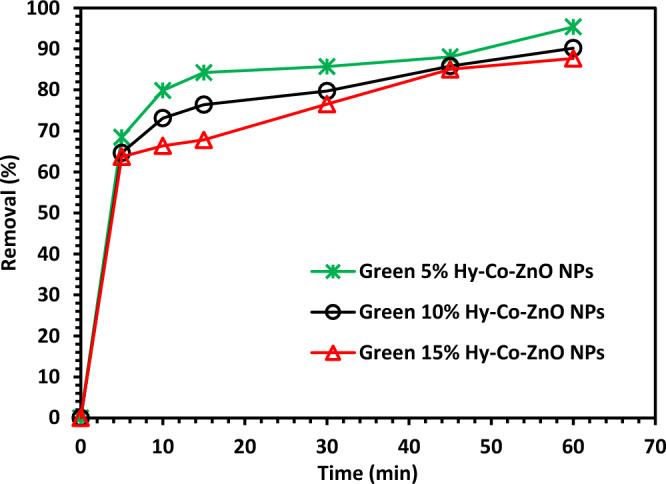
Photocatalytic test of CIPF with Green Hy-ZnO, 5, 10, and 15% Hy-Co-ZnO NPs.

According to the performance, it was shown that Green 5% Hy-Co-ZnO NPs gave the best performance with 99% removal of CIPF after 120 min as shown in Fig. 10.

#### *Point of zero charge (pH*_*PZC*_*) of Green 5% Hy-Co-ZnO NPs*

**Figure 11:**
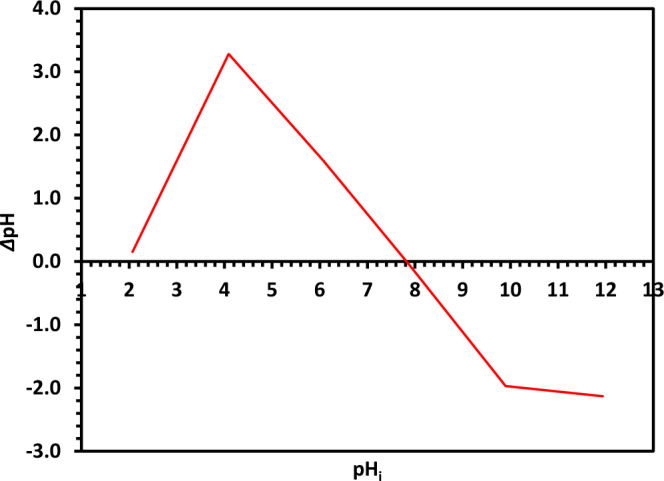
The pH_ZPC_ determination of the Green 5% Hy-Co-ZnO NPs.

To determine the pH_PZC_ of Green 5% Hy-Co-ZnO NPs, the pH drift method was used by using electrolyte solution NaCl (0.1N), whose pH was in steps between 2 and 12 by using 0.1N NaOH and 0.1N HCl. 50 mL of NaCl (0.1N) was taken in the conical flask with 0.10 g of Green 5% Hy-Co-ZnO NPs. To achieve pH equilibrium, the solution control was put on a mechanical shaker for 24 h at 150 rpm^60^. Graphs were then plotted the difference between final and initial pH (∆pH) against initial pH (pH_i_), and the point of interaction of the curve with x aces shows pH_ZPC_^60^. Figure 11 shows that Green 5% Hy-Co-ZnO NPs had a 7.8 pH_PZC_.

#### The pH impact on CIPF photo-degradation.

**Figure 12:**
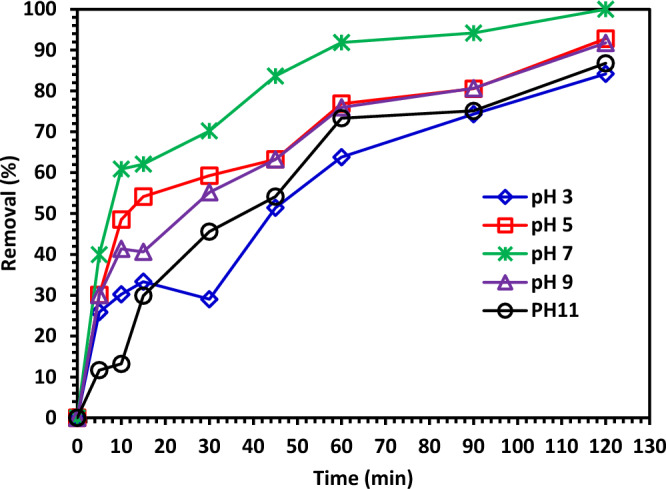
The pH solution impact on CIPF photodegradation using Green 5% Hy-Co-ZnO NPs.

To investigate the impact of the initial solution pH on the photocatalytic degradation of CIPF, the solution pH was adjusted to 3, 5, 7, 9, and 11. The results in Fig. 12 show that the pH of nature (pH = 7) is ideal for the breakdown of CIPF. The initial pH's complicated impact on the photocatalytic destruction of pollutants is dependent on the type of pollutant and the pH_ZPC_ of the photocatalyst, among other factors. The solution pH affects the photocatalyst's surface charge characteristics, which has an important impact on the electrostatic interaction between the pollutant molecules and the catalyst surface. By comparing the characteristics of the catalyst and antibiotic at various pH levels, it is possible to analyze how pH affects the breakdown of CIPF antibiotics. The zero-point charge for Green 5% Hy-Co-ZnO NPs is 7.8, and as a result, the surface of Green 5% Hy-Co-ZnO NPs is positively charged at pH < 7.8 and negatively charged at pH > 7.8^8^. In contrast, CIPF has pka values of 6.09 and 8.2. The adsorption on the surface of Green 5% Hy-Co-ZnO NPs is constrained because, at acidic pH, both Green 5% Hy-Co-ZnO NPs and CIPF are positively charged. The surface of Green 5% Hy-Co-ZnO NPs has a positive surface whereas the surface of CIP is negative at pH values greater than 6.09, which leads to the antibiotic adsorption on the surface of Green 5% Hy-Co-ZnO NPs and an increase in the rate of degradation. When a solution's pH is more than 7.8, CIPF will take on an anionic form (CIP-O^-^), which prevents species from oxidizing and ultimately reduces the effectiveness of CIPF removal^8^.

#### The effect of Green 5% Hy-Co-ZnO dosage on CIPF photodegradation

**Figure 13:**
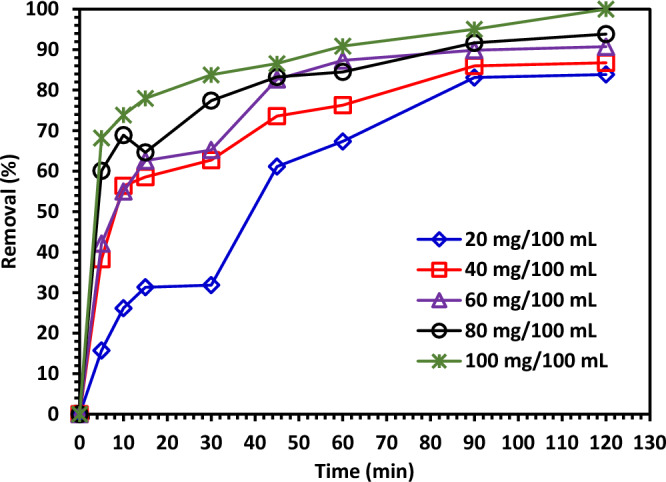
The effect of Green 5% Hy-Co-ZnO NPs dosage on CIPF photodegradation.

Figure 13 depicts the catalyst dosages of the Green 5% Hy-Co-ZnO NPs sample, which fluctuated between 0.2 and 1.0 g/L at intervals of 0.2 g/L. For different amounts of catalyst doses such as 20, 40, 60, 80 and 100 mg/100 mL of solution, it was found that the decomposition rates were 83.8, 86.7, 90.7, 93.8 and 99%, respectively. Because of increasing active surface area being available for redox reactions as the catalyst dose rose, the degradation percentage increased as well^61,62^. For example, during photocatalytic reactions, more charge carriers are produced, and these charge carriers then start the redox reactions. The rate of reaction and degradation % both somewhat decreased with excess catalyst dose. Higher catalyst doses cause the solution's turbidity to increase, which has the effect of aggregating particles and blocking light. As a result, the catalyst's capacity to absorb light is compromised, and the reaction's rate is slowed down^63^.

#### The impact of initial CIPF concentration on its photodegradation

**Figure 14:**
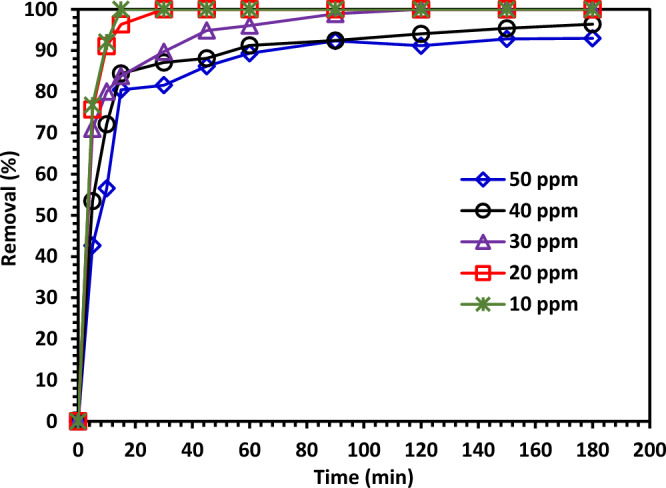
The effect of initial CIPF concentration on its photodegradation.

A series of studies were conducted at catalyst dose (1.0 g/L) at pH 7 using varied CIPF starting concentrations (C_0_ = 10, 20, 30, 40, and 50 ppm) to study the influence of initial CIPF concentration on the photocatalytic efficiency of Green 5% Hy-Co-ZnO NPs. Figure 14 demonstrates a definite decrease in degradation efficiency with an increase in CIPF initial concentration. According to the results, the saturation of the Co-ZnO surface may be what prevents CIPF photodegradation at higher initial concentrations. This is because there would be more competition for the active sites at higher CIPF concentrations, which would lead to fewer photons reaching the catalyst surface. As a result, fewer hydroxyl radicals (**·**OH) and electron–hole pairs are generated^8^.

#### The effect of shaking speed on the photodegradation of CIPF

**Figure 15:**
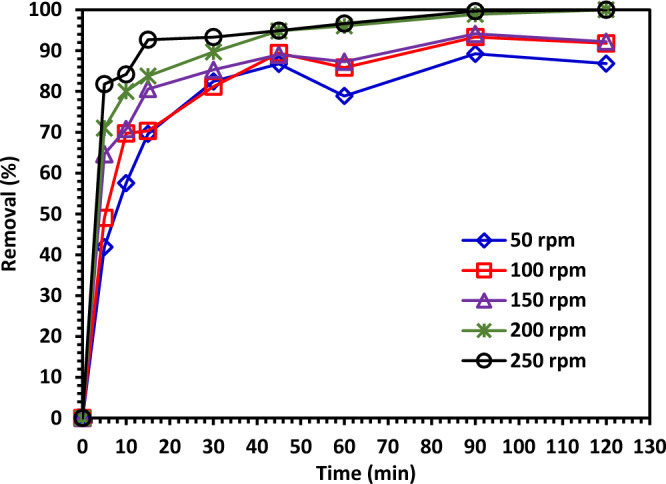
The impact of shaking speed on the photodegradation of CIPF using Green 5% Hy-Co-ZnO NPs.

The impact of shaking speed was examined on the photodegradation of CIPF using various shaking speeds between 50 and 250 rpm. Continuous shaking is crucial for creating a homogenous solution out of the antibiotic solution and photocatalysts and for enhancing solution flow along the surfaces of the photocatalysts. Figure 15 shows the effects of shaking speed on photocatalytic efficiency for a catalyst under a visible light source. The efficiency of Green 5% Hy-Co-ZnO NPs to degrade was observed to increase with faster shaking. Shaking may increase the combination of photocatalysts and antibiotics in water and localized turbulence near the photocatalyst base. This reduces the distance of the boundary layer and increases the efficiency of the photo-degradation of antibiotics in water^64^.

#### The temperature impact on the CIPF photodegradation

**Figure 16:**
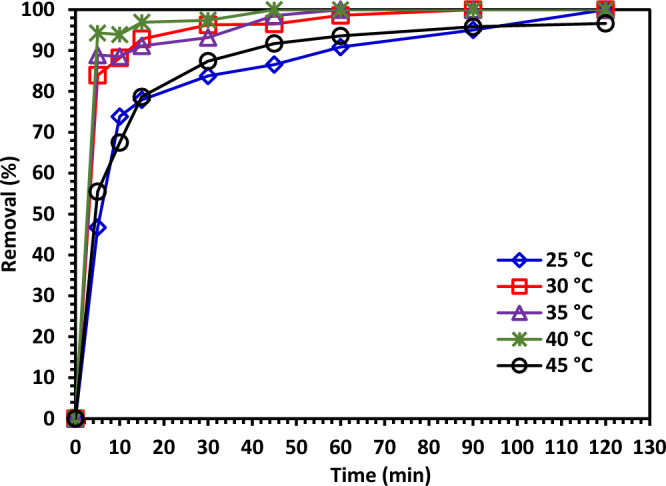
The temperature impact on the photodegradation of CIPF using Green 5% Hy-Co-ZnO NPs.

Experiments series were performed to investigate the impact of temperature on the photodegradation rate of ciprofloxacin using Green 5% Hy-Co-ZnO NPs at temperatures ranging from 25 to 45 °C. Figure 16 shows the temperature impact on the photocatalytic degradation rate of ciprofloxacin at a fixed starting concentration of 30 ppm and 1.0 g/L of Green 5% Hy-Co-ZnO NPs. Figure 16 indicates that the photocatalytic degradation rate of ciprofloxacin increases with an increase of temperature due to increasing temperature causing to increase in the generated free radicals, which leads to a decrease in the recombination process^65^. The degradation kinetics slowed down up to 45 °C as the temperature rose. The fact that fluoroquinolones become more stable after being exposed to heat stress may be one explanation.

#### The scavengers' impact on the removal efficiency

**Figure 17:**
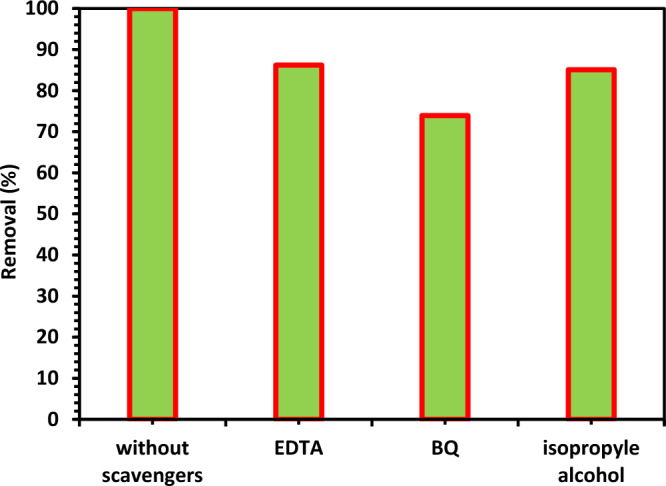
Effect of diverse scavengers on photodegradation of CIPF in the existence of 1.0 g/L of Green 5% Hy-Co-ZnO NPs catalyst at reaction pH = 7.

A trapping experiment was used to identify the active species in the photocatalytic reaction. Following the addition of three different free radical scavengers, namely BQ, IPA, and EDTA, to the photocatalytic degradation system, Fig. 17 displays the rate of ciprofloxacin degradation. BQ, IPA and EDTA were applied to capture ^−^O_2_^**.**^, OH and h^+^, respectively. With the addition of BQ, the degradation rate decreased to some extent, demonstrating that ^-^O_2_^**.**^ had a particular impact on CIPF degradation. With the addition of EDTA, the effectiveness of ciprofloxacin's degradation was similarly decreased, indicating that h^+^ played a part in the reaction process^31^. The effectiveness of ciprofloxacin's photodegradation was considerably decreased when IPA was added as a scavenger of ^**.**^OH, demonstrating the primary role of ^**.**^OH in this process. These findings suggest that ^**.**^OH plays a significant role in the photocatalytic degradation of ciprofloxacin, which occurs in the order^**.**^OH > h^+^  > ^−^O_2_^**.**^^31^.

#### Kinetics of CIPF photodegradation

**Figure 18:**
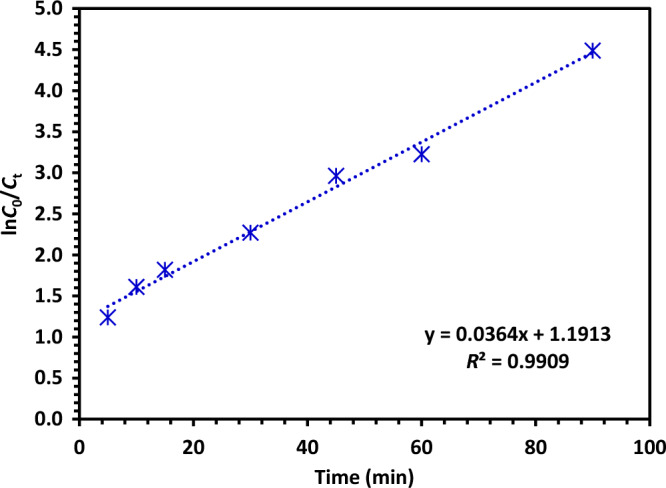
Kinetics plot for the photocatalytic degradation efficiency of CIPF using Green 5% Hy-Co-ZnO NPs.

Experimental data were utilized to create a pseudo-first-order model (Eq. 10) that allows for a more detailed analysis of the photocatalytic reaction kinetics of the catalyst's role in CIPF degradation^66^.

10 $$ln\frac{{C}_{0}}{{C}_{t}}=kt$$

where *k* stands for the rate constant, *C*_0_ stands for the CIPF beginning concentration, C_t_ stands for the CIPF final concentration. Figure 18 depicts the analysis of the CIPF's time-dependent changes and its kinetics. The *k* value was calculated from the slope of the plot of ln*C*_0_/*C*_t_ against reaction time (*t*), as presented in Fig. 18. As a result, the provided method was applied to define the CIPF rate constants, which came out to be 0.0364 min^−1^. This suggests that pseudo-first-order kinetics governs the degrading process.

#### Photodegradation mechanism of CIPF using Green 5% Hy-Co-ZnO NPs

Electrons (*e*) in the conduction band assisted in the production of superoxide free radicals (^.^O_2_^–^) (Fig. 1). Because reactive oxygen species (ROS) are so highly reactive, they were joined to create the H_2_O_2_ molecule. The H_2_O_2_ molecule was broken down into ^.^OH free radicals, which caused the ciprofloxacin to be broken down. Below, in Eqs. (11–16), are the chemical formulas for ciprofloxacin degradation through active species.

11 $$ {\text{Co}} - {\text{ZnO}} + h\upsilon \to {\text{h}}^{ + } \left( {{\text{VB}}} \right) + {\text{e}}^{-} \left( {{\text{CB}}} \right) $$

12 $$ {\text{h}}^{ + } \left( {{\text{VB}}} \right) + {\text{H}}_{{2}} {\text{O}} \to {\text{H}}^{ + } +^{ \cdot } {\text{OH}} $$

13 $$ {\text{e}}^{-} \left( {{\text{CB}}} \right) + {\text{O}}_{{2}} \to {\text{CB }} +^{.} {\text{O}}_{{2}}^{-} $$

14 $$^{.} {\text{O}}_{{2}}^{-} + {\text{ H}}^{ + } + {2}^{.} {\text{OH}} \to {\text{O}}_{{2}} + {\text{2 H}}_{{2}} {\text{O}}_{{2}} $$

15 $$ {\text{2H}}_{{2}} {\text{O}}_{{2}} \to {4}^{.} {\text{OH}} $$

16 $$ ^{.} {\text{O}}_{{2}}^{-} +^{.} {\text{OH}} + {\text{CIPF}} \to {\text{CO}}_{{2}} + {\text{H}}_{{2}} {\text{O}} $$

#### Reusability of photocatalyst

**Figure 19:**
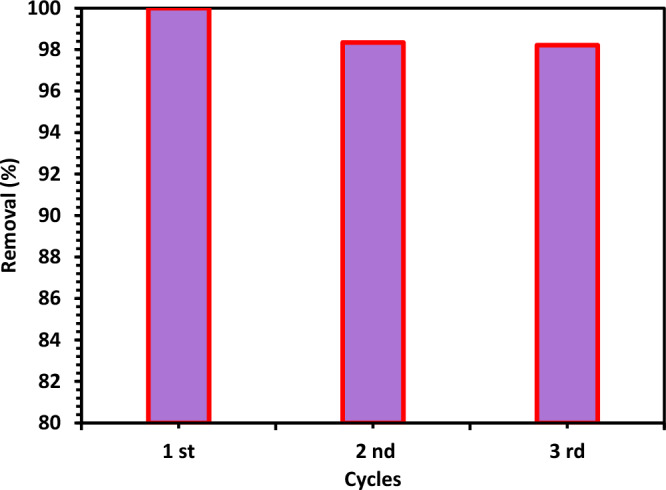
Recyclability of Green 5% Hy-Co-ZnO NPs within three consecutive cycles for the photodegradation of CIPF.

A study of the reproducible stability of the prepared Green 5% Hy-Co-ZnO NPs is important for proving the catalyst's effectiveness and affordability. After each CIPF photodegradation experiment, the catalyst was separated, repeatedly cleaned with DW and EtOH, and then re-used for more research. As can be observed in Fig. 19, the synthesized photocatalyst's photocatalytic activity remained essentially consistent throughout three cycles for the photodegradation of CIPF. In these investigations, the Green 5% Hy-Co-ZnO NPs exhibit great reusability and high photostability, with small deactivation.

#### Modeling and statistical analysis

Design Expert (13.0.5.0) software was used to analyze the impact of various parameters on the effectiveness of the CIPF degradation process, and experiments were planned to use the CCD to optimize the key operating parameters and maximize the antibiotic's photodegradation rate in aqueous solutions. Table 4 displays the CCD matrix and experimental findings from the photocatalytic degradation runs. The dependent and independent variables were correlated using the second-order polynomial response Eq. (17)^39^.

**Table 4:** The CCD table for CIPF photodegradation includes intended values as well as experimental and anticipated findings.

run	A Catalyst dosage	B Antibiotic dosage	C Shaking speed	D PH	Removal Actual value	Removal Predicted value
1	60	30	250	7	91.03	88.51
2	100	30	150	7	91.29	88.95
3	40	40	200	9	91.56	82.67
4	60	30	150	7	94.41	94.41
5	80	20	200	5	70.61	74.01
6	80	40	100	5	96.42	87.95
7	80	20	200	9	87.69	81.34
8	80	20	100	5	57.50	64.65
9	60	30	150	11	69.22	75.05
10	60	30	150	7	94.41	94.41
11	60	30	150	7	94.41	94.41
12	80	40	200	9	92.08	91.89
13	40	20	200	5	71.05	66.65
14	40	40	100	5	86.64	91.26
15	40	40	100	9	90.07	90.29
16	60	30	150	7	94.41	94.41
17	40	40	200	5	84.09	87.35
18	60	50	150	7	94.70	95.04
19	60	30	150	7	94.41	94.41
20	20	30	150	7	78.25	78.69
21	60	30	50	7	86.16	86.78
22	80	20	100	9	75.32	75.69
23	60	30	150	7	94.41	94.41
24	60	10	150	7	59.09	56.84
25	40	20	100	9	78.15	68.44
26	40	20	100	5	59.78	63.61
27	80	40	100	9	90.52	93.19
28	80	40	200	5	82.37	90.36
29	60	30	150	3	76.43	68.69
30	40	20	200	9	55.66	67.77

17 $$Y={\beta }_{0}+{\sum }_{i=1}^{k}{\beta }_{i}{x}_{i}+{\sum }_{i=1}^{k}{\beta }_{ii}{x}_{i}^{2}+{\sum }_{i<1}^{k}{\beta }_{ij}{x}_{i}{x}_{j}+\varepsilon $$

In this countenance, *β*_0_ represents the interception term, *x*_i_ and *x*_j_ are the variable parameters of *i* and *j* with the number *k*, and *β*_i_, *β*_ii_, and *β*_ij_ represent the linear, quadratic, and interaction parameter coefficients, respectively. Lastly, the term *ɛ* indicates the random error. An empirical link between the response and independent parameters for CIPF (Y) as in Eq. (18) is based on these findings.

18 $$ {\text{Y}} = {94}.{41} + {2}.{\text{57A}} + {9}.{\text{55B}} + 0.{\text{4344C}} + {1}.{\text{59D}} - {1}.0{\text{9AB}} + {1}.{\text{58AC}} + {1}.{\text{55AD}} - {1}.{\text{74BC}} - {1}.{\text{45BD}} - 0.{\text{9281CD}} - {2}.{\text{65A}}^{{2}} - {4}.{\text{62B}}^{{2}} - {1}.{\text{69C}}^{{2}} - {5}.{\text{63D}}^{{2}} $$

#### ANOVA regression analysis

The ANOVA analysis was used to demonstrate how important model parameters affected the results. Additionally computed were the degrees of freedom (dF), the sum of squares, and the mean sum of squares. High relevance for a parameter is exposed by a higher F-value and a lower P-value (0.05)^67–76^. The amount of CIPF antibiotic dose is most important, followed by the interaction between CIPF dosage and pH, which are both unimportant characteristics, according to Table S4. The F-value and P-value of the model are 5.67 and 0.0009, respectively, which support its importance.

Equation (6) depicts the model in terms of the process parameters. The response variable is known to be affected positively by factors with positive coefficients and negatively by variables with negative coefficients^68^. The most utilized parameter, the determination coefficient (*R*^2^), helps the suggested model via ANOVA to confirm its statistical significance and suitability. The *R*^2^ between actual and theoretical measures is what determines it^77^. The current model's *R*^2^ value of 84.12% illustrates the model's reliability. This suggests that process variables can account for 84.12% of variability in photodegradation. The adjusted coefficient of determination (adj. *R*^2^) is computed for a more thorough study. The adj. *R*^2^ is ensured to be decreased by duplicate variables^77^. The adj. *R*^2^ in this instance is 69.29%, highlighting the model's importance.

#### Validation of the response surface model

Here, the model's accuracy was assessed using a predicted versus residuals values diagram and a normal plot of residuals. The residues are situated on or close to the usual line, as shown in Fig. S4a, demonstrating that the residuals are normal. The residuals are widely dispersed around the baseline and lack a clear pattern, as seen by the predicted graph (Fig. S4b). The closeness of the predicted values to the actual values is confirmed by Fig. S4c. All three designs demonstrate the model's suitability.

The perturbation plot, which is depicted in Fig. S4d, was created to investigate the simultaneous impact of four variables on antibiotic clearance. This plot, in general, compares the influences of all components at a certain location in the design space^78^. The regulating factors for CIPF degradation were the following: pH level (D), shaking rate (C), catalyst dosage (A), and CIPF antibiotic dosage (B). Therefore, the fact that the curve for CIPF antibiotic dosage exists shows that this component has a greater impact than the others.

#### The response variables analysis (3-D response surface plot)

**Figure 20:**
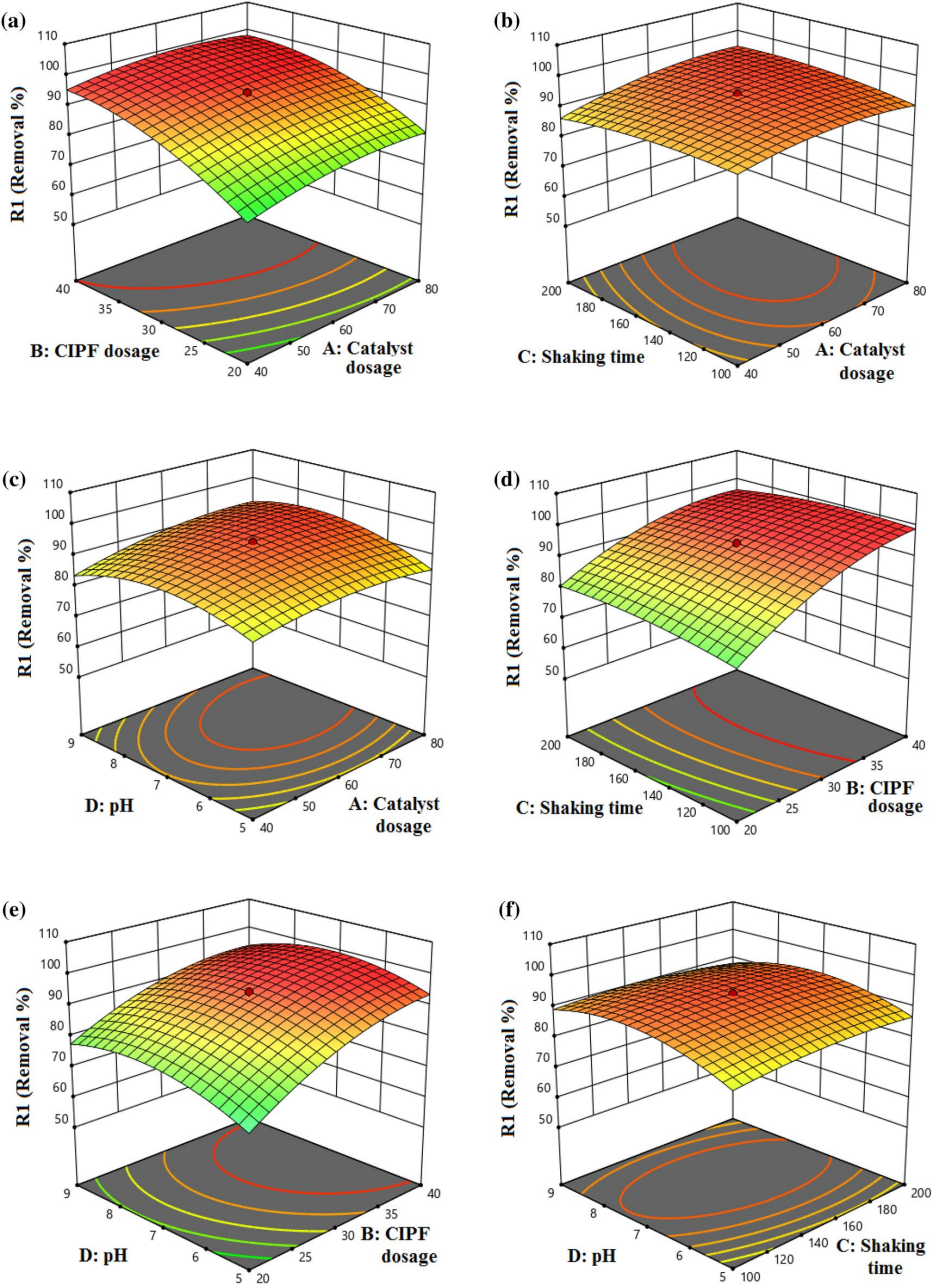
3D response surface plots, (**a**) CIPF antibiotic dosage and Green 5% Hy-Co-ZnO NPs catalyst dosage, (**b**) Shaking speed and Green 5% Hy-Co-ZnO NPs catalyst dosage, (**c**) pH value and Green 5% Hy-Co-ZnO NPs catalyst dosage, (**d**) CIPF antibiotic dosage and shaking speed, (**e**) pH value and CIPF antibiotic dosage, (**f**) pH value and shaking speed.

The RSM-CCD suggested model function and the 3-D response surface plots are useful tools for the graphical representation of the interaction impacts on the factors of the response values. Figure 20a shows the 3-D response surface plot based on the interaction impact of two significant factors, the dose of antibiotics and the amount of Green 5% Hy-Co-ZnO NPs catalyst on the removal of CIPF by the Green 5% Hy-Co-ZnO NPs photocatalyst. Consequently, the CIPF removal by the photolysis process was increased by increasing the catalyst dosage due to the higher production of electron–hole pairs^66^. On the 3-D response surface plot shown in Fig. 20b, the interaction effects of two significant parameters, the dose of Green 5% Hy-Co-ZnO NPs catalyst and shaking speed, are displayed. As the shaking speed increased, the photodegradation of CIPF increased by increasing sample loading up to approximately 70 mg, according to the data. The photocatalytic activity decreased when the sample loading was raised. Despite the shaking's speed rising. The excess catalyst (> 70 mg) acting as a recombination center was the cause of this.^66^. The interaction effects between 5% Co-ZnO dose range of 40–80 mg and pH of medium in the range of 5–9 is shown in Fig. 20c. The catalyst's pH_PZC_ is 7.8, and the best reaction was produced when the pH was between 5 and 7. On the other hand, more active sites, ^**.**^OH or ^**.**^O_2_ radicals, and (e^-^/h^+^) formed pairs were produced on the photocatalyst surface when the catalyst dose was increased from 40 to 80 mg^71^. Thus, improved photolysis yield of CIPF particles in the above ranges was obtained. Figure 20d shows how the photocatalytic degradation efficiency of CIPF was affected by antibiotic dose and shaking speed, which increased as the shaking speed increased. Figure 20e depicts the contour plot of the conversion percentage as a function of pH solution and CIPF concentration, all other factors being held constant (catalyst dosage and shaking speed). More CIPF molecules are formed on the catalyst surface when pH increases from 5.0 to 7.0 due to a decrease in repulsive force. In addition, increasing the concentration of CIPF enhanced the probability of collisions between the molecules of CIPF and the radicals on the catalyst's surface. Since OH and O_2_ radicals have a relatively short lifespan, they should be consumed right away wherever they are formed^78^. Figure 20f shows how pH and shaking speed affect the effectiveness of CIPF's photocatalytic degradation, where the optimum reaction was obtained at a pH of around 7 during the entire range of shaking speed (100–200 rpm), then decreased from 7 to 9.

**Figure 21:**
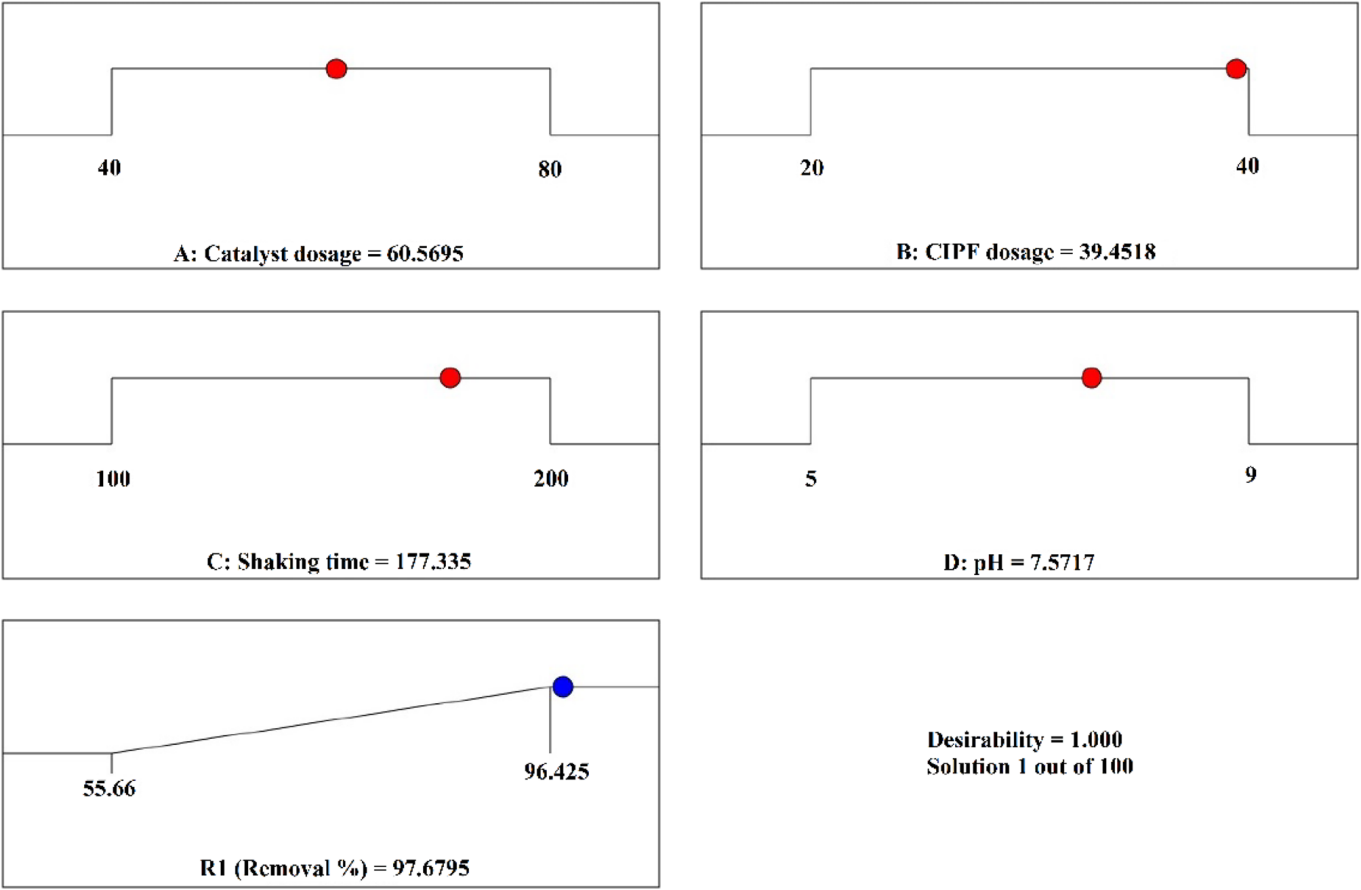
A final answer through CCD with optimized settings.

Figure 21 illustrates the variables' optimum conditions, which were determined as 39.45 ppm CIPF antibiotic dosage, 60.56 mg Green 5% Hy-Co-ZnO NP catalyst dosage, 177.34 rpm shaking speed and 7.54 pH using the desirability function of the model within these conditions: the CIPF removal yield is 96.4%.

## Conclusion

In this study, green Hy-ZnO NPs and cobalt-doped ZnO NPs (5, 10, 15% of Co) were synthesized utilizing a biosynthesis approach helped by hydrothermal method using red algae *P. capillacea* water extract. The synthesized Hy-ZnO NPs and cobalt-doped ZnO NPs were characterized and confirmed using FTIR, XRD, XPS, BET, SEM, EDX, TEM, TGA, and DRS UV–vis spectroscopy. The change in the lattice strain (ε) that arises because of the addition of Co to ZnO was confirmed by XRD. Moreover, the increase in the lattice strain on doping is associated with the increase in dislocation density. The photodegradation of ciprofloxacin by Green 5% Hy-Co-ZnO NPs reached 99% after 120 min as a result of their significant photocatalytic activity towards the antibiotic. The correlation coefficients plot of CIPF represented that the photodegradation of CIPF follows pseudo-first-order kinetics. CCD optimization model of the Green 5% Hy-Co-ZnO NPs was also studied. It has successfully assessed the photocatalytic activity of Green 5% Hy-Co-ZnO NPs toward the removal of CIPF under visible Led-light, and it accomplished ~ 98% by the optimal conditions, and the result revealed that at 5% Hy-Co–ZnO NPs dosage of 60.569 mg, 39.45 mg/L of CIPF primary concentration, pH 7.57, and 177.33 rpm, the removal can reach 96.4% after 120 min.

## Supplementary Information

Supplementary Information.

## Data Availability

Upon request to the corresponding author of the study, the datasets utilized in this inquiry are available for inspection.

## References

[1] Shah, N. S. *et al.* Nano-zerovalent copper as a Fenton-like catalyst for the degradation of ciprofloxacin in aqueous solution. *J. Water Proc. Eng.* **37**, 101325. 10.1016/j.jwpe.2020.101325 (2020).

[2] Sharma, G. *et al.* Fabrication and characterization of trimetallicnano-photocatalyst for remediation of ampicillin antibiotic. *J. Mol. Liq.* **260**, 342–350. 10.1016/j.molliq.2018.03.059 (2018).

[3] Hassaan, M. A. *et al.* Degradation mechanism of Direct Red 23 dye by advanced oxidation processes: A comparative study. *Toxin Rev.* **41**, 38–47 (2022).

[4] Roma, M., Weller, M. & Wentzell, S. *Removal of Ciprofloxacin from Water Using Adsorption UV Photolysis and UV/H*_*2*_*O*_*2*_*Degradation Project Report* (Worcester Polytechnic Institute, 2011).

[5] Lapworth, D. J., Baran, N., Stuart. M. E. & Ward, R. S. Emerging organic contaminants in groundwater: A review of sources, fate and occurrence. *Environ. Pollut*. **163**, 287–303. 10.1016/j.envpol.2011.12.034 (2012). 10.1016/j.envpol.2011.12.034 22306910

[6] Johnson, A. C., Keller, V., Dumont, E. & Sumpter, J. P. Assessing the concentrations and risks of toxicity from the antibiotics ciprofloxacin, sulfamethoxazole, trimethoprim, and erythromycin in European rivers. *Sci. Total Environ.* **511**, 747–755. 10.1016/j.scitotenv.2014.12.055 (2015). 25617699 10.1016/j.scitotenv.2014.12.055

[7] Li, J. *et al.* Study of ciprofloxacin removal by biochar obtained from used tealeaves. *J. Environ. Sci.* **73**, 20–30. 10.1016/j.jes.2017.12.024 (2018). 10.1016/j.jes.2017.12.024 30290868

[8] Aghdasi, S. & Shokri, M. Photocatalytic degradation of ciprofloxacin in the presence of synthesized ZnO nanocatalyst: The effect of operational parameters. *Iran. J. Catal.* **6**, 481–487 (2016).

[9] Liu, Y. *et al.* Design and structural of Sm-doped SbFeO_3_ nanopowders and immobilized on poly (ethylene oxide) for efficient photocatalysis and hydrogen generation under visible light irradiation. *Surf. Interfaces* **26**, 101292 (2021).

[10] Bahadoran, A. *et al.* Fabrication and structural of gold/cerium nanoparticles on tin disulfide nanostructures and decorated on hyperbranched polyethyleneimine for photocatalysis, reduction, hydrogen production and antifungal activities. *J. Photochem. Photobiol. A* **416**, 113316 (2021).

[11] Al-Namshah, K. S., Shkir, M., Ibrahim, F. A. & Hamdy, M. S. Auto combustion synthesis and characterization of Co doped ZnO nanoparticles with boosted photocatalytic performance. *Physica B* **625**, 413459. 10.1016/j.physb.2021.413459 (2022).

[12] Wang, J., Sun, J., Huang, J., Fakhri, A. & Gupta, V. K. Synthesis and its characterization of silver sulfide/nickel titanate/chitosan nanocomposites for photocatalysis and water splitting under visible light, and antibacterial studies. *Mater. Chem. Phys.* **272**, 124990 (2021).

[13] Tanji, K. *et al.* Design and simulation of a photocatalysis reactor for rhodamine B degradation using cobalt-doped ZnO film. *React. Kinet. Mech. Catal.* **134**, 1017–1038. 10.1007/s11144-021-02116-3 (2021).

[14] Liu, B. & Zeng, H. C. Hydrothermal synthesis of ZnO nanorods in the diameter regime of 50 nm. *J. Am. Chem. Soc.* **125**, 4430–4431. 10.1021/ja0299452 (2003). 12683807 10.1021/ja0299452

[15] Rezaei, M. & Nezamzadeh-Ejhieha, A. The ZnO-NiO nano-composite: A brief characterization, kinetic and thermodynamic study and study the Arrhenius model on the sulfasalazine photodegradation. *Int. J. Hydrog. Energy* **45**, 24749–24764. 10.1016/j.ijhydene.2020.06.258 (2020).

[16] Chakma, S. & Moholkar, V. S. Investigation in mechanistic issues of sonocatalysis and sonophotocatalysis using pure and doped photocatalysts. *Ultrason. Sonochem.* **22**, 287–299. 10.1016/j.ultsonch.2014.06.008 (2015). 24986798 10.1016/j.ultsonch.2014.06.008

[17] Lu, Y. *et al.* A high performance cobalt doped ZnO visible light photocatalyst and its photogenerated charge transfer properties. *Nano Res.* **4**, 1144–1152. 10.1007/s12274-011-0163-4 (2011).

[18] Hassaan, M. A. *et al.* Enhancement of biogas production via green ZnO nanoparticles: Experimental results of selected herbaceous crops. *Chem. Eng. Commun.* **208**, 242–255. 10.1080/00986445.2019.1705797 (2021).

[19] Amirante, R. *et al.* Effects of ultrasound and green synthesis ZnO nanoparticles on biogas production from Olive Pomace. *Energy Procedia* **148**, 940–947. 10.1016/j.egypro.2018.08.091 (2018).

[20] Hassaan, M., El Katory, M., Ali, R. M. & El Nemr, A. Photocatalytic degradation of reactive black 5 using Photo-Fenton and ZnO nanoparticles under UV irradiation. *Egypt. J. Chem.* **63**, 1443–1459. 10.21608/ejchem.2019.15799.1955 (2020).

[21] El Nemr, A., Hassaan, M. A., Elkatory, M. R., Ragab, S. & Pantaleo, A. Efficiency of Fe_3_O_4_ nanoparticles with different pretreatments for enhancing biogas yield of macroalgae ulva intestinalis linnaeus. *Molecules* **26**, 5105. 10.3390/molecules26165105 (2021). 34443690 10.3390/molecules26165105 PMC8399479

[22] Hassaan, M. A. *et al.* Techno-economic analysis of ZnO nanoparticles pretreatments for biogas production from barley straw. *Energies* **13**, 5001. 10.3390/en13195001 (2020).

[23] Sharma, D., Kanchi, S. & Bisetty, K. Biogenic synthesis of nanoparticles: A review. *Arab. J. Chem.* **12**, 3576–3600. 10.1016/j.arabjc.2015.11.002 (2019).

[24] Siddiquee, M. A. *et al.* Biogenic synthesis, in-vitro cytotoxicity, esterase activity and interaction studies of copper oxide nanoparticles with lysozyme. *J. Mater. Res. Technol.* **13**, 2066–2077. 10.1016/j.jmrt.2021.05.078 (2021).

[25] Arumugam, V., Subramaniam, S. & Krishnan, V. Green synthesis and characterization of zinc oxide nanoparticles using *Berberis tinctoria Lesch.* leaves and fruits extract of multi-biological applications. *Nanomed. Res. J.* **6,** 128–147. 10.22034/nmrj.2021.02.005 (2021).

[26] Aldhalmi, A. K. *et al.* A novel fabricate of iron and nickel-introduced bimetallic MOFs for quickly catalytic degradation via the peroxymonosulfate, antibacterial efficiency, and cytotoxicity assay. *Inorg. Chem. Commun.* **153**, 110823 (2023).

[27] Syed, A. *et al.* Highly-impressive performances of novel NiCo_2_O_4_/Bi_2_O_3_/Ag_2_ZrO_3_ nanocomposites in photocatalysis system, removal pathway, toxicity estimation, and antibacterial activities. *J. Taiwan Inst. Chem. Eng.* **149**, 105004 (2023).

[28] Sharma, D. *et al.* Biosynthesis of hematite nanoparticles using Rheum emodi and their antimicrobial and anticancerous effects in vitro. *J. Photochem. Photobiol.* **206**, 111841. 10.1016/j.jphotobiol.2020.111841 (2020). 10.1016/j.jphotobiol.2020.111841 32197209

[29] Sundrarajan, M., Ambika, S. & Bharathi, K. Plant-extract mediated synthesis of ZnO nanoparticles using Pongamia pinnata and their activity against pathogenic bacteria. *Adv. Powder Technol.* **26**, 1294–1299. 10.1016/j.apt.2015.07.001 (2015).

[30] Haque, M. J., Bellah, M. M., Hassan, M. R. & Rahman, S. Synthesis of ZnO nanoparticles by two different methods & comparison of their structural, antibacterial, photocatalytic and optical properties. *Nano Express.* **1**, 010007. 10.1088/2632-959X/ab7a43 (2020).

[31] Hassaan, M. A. *et al.* Dual action of both green and chemically synthesized zinc oxide nanoparticles: Antibacterial activity and removal of Congo red dye. *Desalin. Water Treat.* **218**, 423–435. 10.5004/dwt.2021.26988 (2021).

[32] Letsholathebe, D., Thema, F. T., Mphale, K., Maabong, K. & Magdalane, C. M. Green synthesis of ZnO doped Moringa oleifera leaf extract using Titon yellow dye for photocatalytic applications. *Mater. Today: Proc.* **36**, 475–479. 10.1016/j.matpr.2020.05.119 (2021).

[33] El Nemr, A., El-Sikaily, A., Khaled, A. & Abdelwahab, O. Removal of toxic chromium from aqueous solution, wastewater, and saline water by marine red alga *Pterocladia capillacea* and its activated carbon. *Arab. J. Chem.* **8**, 105–117. 10.1016/j.arabjc.2011.01.016 (2015).

[34] Hassaan, M. A. *et al.* Synthesis, characterization, and synergistic effects of modified biochar in combination with α-Fe_2_O_3_ NPs on biogas production from red algae *Pterocladia capillacea*. *Sustainability* **13**, 9275. 10.3390/su13169275 (2021).

[35] Yaacob, N. A., Khasri, A., Ridzuan, M. J. M., Salleh, N. H. M. & Chaijak, P. *Synergistic Adsorption/Photodegradation of Ciprofloxacin by UV light-driven Nanocomposite Photocatalyst of Cu doped AC/TiO*_*2*_. *Experimental design via RSM-CCD*. 10.21203/rs.3.rs-2984198/v1 (2023).

[36] Hasanpour, M., Motahari, S., Jing, D. & Hatami, M. Statistical analysis and optimization of photodegradation efficiency of methyl orange from aqueous solution using cellulose/zinc oxide hybrid aerogel by response surface methodology (RSM). *Arab. J. Chem.* **14**, 103401. 10.1016/j.arabjc.2021.103401 (2021).

[37] Nasiri, A., Malakootian, M., Shiri, M. A., Yazdanpanah, G. & Nozari, M. CoFe_2_O_4_@methylcellulose synthesized as a new magnetic nanocomposite to tetracycline adsorption: Modeling, analysis, and optimization by response surface methodology. *J. Polym. Res.*10.1007/s10965-630021-02540-y (2021).

[38] Chawla, H. *et al.* Visible LED-light driven photocatalytic degradation of organochlorine pesticides (2, 4-D & 2, 4-DP) by Curcuma longa mediated bismuth vanadate. *J. Clean. Prod.* **367**, 132923. 10.1016/j.jclepro.2022.132923 (2022).

[39] Taoufik, N. *et al.* MgO-La_2_O_3_ mixed metal oxides heterostructure catalysts for photodegradation of dyes pollutant: Synthesis, characterization and artificial intelligence modelling. *Environ. Sci. Pollut. Res.* **30**, 23938–32396. 10.1007/s11356-022-23690-6 (2023). 10.1007/s11356-022-23690-6 36329247

[40] Yılmaz, M. *et al.* The use of Mandarin-Biochar-O3-TETA (MBT) produced from Mandarin peels as a natural adsorbent for the removal of Acid Red 35 (AR35) dye from water. *Environ Process* **9**, 1–32 (2022).

[41] Sarkar, S. & Sarkar, R. Synthesis, characterization and tribological study of zinc oxide nanoparticles. *Mater. Today Proc.* **44**, 3606–3612. 10.1016/j.matpr.2020.09.595 (2021).

[42] Naik, E. I., Naik, H. B., Sarvajith, M. S. & Pradeepa, E. Co-precipitation synthesis of cobalt doped ZnO nanoparticles: Characterization and their applications for biosensing and antibacterial studies. *Inorg. Chem. Commun*. **130**, 108678. 10.1016/j.inoche.2021.108678 ‏(2021).

[43] Mustafa, S. M., Barzinjy, A. A. & Hamad, A. H. An environmentally friendly green synthesis of Co^2+^ and Mn^2+^ ion doped ZnO nanoparticles to improve solar cell efficiency. *J. Environ. Chem. Eng.* **11**, 109514. 10.1016/j.inoche.2021.108678 (2023).

[44] Elilarassi, R. & Chandrasekaran, G. Structural, optical and magnetic properties of nanoparticles of ZnO:Ni-DMS prepared by sol–gel method. *Mater. Chem. Phys.* **123**, 450–455. 10.1016/j.matchemphys.2010.04.039 (2010).

[45] Kalia, R. *et al.* New insights on photocatalytic hydrogen evolution of ZnFe_2_− xGaxO_4_ (0 ≤x ≤ 2) solid solutions: Role of oxygen vacancy and ZnO segregated phase. *J. Alloys Compd.* **875**, 159905. 10.1016/j.jallcom.2021.159905 (2021).

[46] Parchur, A. K. & Ningthoujam, R. S. Preparation and structure refinement of Eu^3+^ doped CaMoO_4_ nanoparticles. *Dalton Trans.* **40**, 7590–7594. 10.1039/c1dt10327j (2011). 21695334 10.1039/c1dt10327j

[47] Ntwaeaborwa, O. M. *et al.* Structural, optical and photoluminescence properties of Eu^3+^ doped ZnO nanoparticles. *Spectrochim. Acta A* **182**, 42–49. 10.1016/j.saa.2017.03.067 (2017). 10.1016/j.saa.2017.03.067 28391073

[48] Cun, T., Dong, C. & Huang, Q. Ionothermal precipitation of highly dispersive ZnO nanoparticles with improved photocatalytic performance. *Appl. Surf. Sci.* **384**, 73–82. 10.1016/j.apsusc.2016.05.008 (2016).

[49] Filip, J. *et al.* pH-switchable interaction of a carboxybetaine ester-based SAM with DNA and gold nanoparticles. *Langmuir* **33**, 6657–6666. 10.1021/acs.langmuir.7b00568 (2017). 28628328 10.1021/acs.langmuir.7b00568

[50] Dung, T. M. *et al.* Influence of surfactant on the preparation of silver nanoparticles by polyol method. *Adv. Nat. Sci. Nanosci. Nanotechnol.* **3**, 035004. 10.1088/2043-6262/3/3/035004 (2012).

[51] Nguyen, N. T., Nguyen, N. T. & Nguyen, V. A. In situ synthesis and characterization of ZnO/chitosan nanocomposite as an adsorbent for removal of Congo red from aqueous solution. *Adv. Polym. Technol.*10.1155/2020/3892694 (2020).

[52] Farhadian, N. *et al.* Chitosan modified N, S-doped TiO_2_ and N, S-doped ZnO for visible light photocatalytic degradation of tetracycline. *Int. J. Biol. Macromol.* **132**, 360–373. 10.1016/j.ijbiomac.2019.03.217 (2019). 30940592 10.1016/j.ijbiomac.2019.03.217

[53] Zheng, Y., Cheng, B., You, W., Yua, J. & Ho, W. 3D hierarchical graphene oxide-NiFe LDH composite with enhanced adsorption affinity to Congo red, methyl orange and Cr(VI) ions. *J. Hazard. Mat.* **369**, 214–225. 10.1016/j.jhazmat.2019.02.013 (2019). 10.1016/j.jhazmat.2019.02.013 30776604

[54] Khalid, A. *et al.* Enhanced optical and antibacterial activity of hydrothermally synthesized cobalt-doped zinc oxide cylindrical microcrystals. *Materials* **14**, 3223. 10.3390/ma14123223 (2021). 34207950 10.3390/ma14123223 PMC8230675

[55] Vindhya, P. S., Suresh, S., Kunjikannan, R. & Kavitha, V. T. Antimicrobial, antioxidant, cytotoxicity and photocatalytic performance of Co doped ZnO nanoparticles biosynthesized using Annona Muricata leaf extract. *J. Environ. Health Sci. Eng.*10.1007/s40201-023-00851-4 (2023). 37159742 10.1007/s40201-023-00851-4 PMC10163207

[56] Anandan, M., Dinesh, S., Krishnakumar, N. & Balamurugan, K. Influence of Co doping on combined photocatalytic and antibacterial activity of ZnO nanoparticles. *Mater. Res. Express* **3**, 115009. 10.1088/2053-1591/3/11/115009 (2016).

[57] Poornaprakash, B., Chalapathi, U., Subramanyam, K., Vattikuti, S. P. & Park, S. H. Wurtzite phase Co-doped ZnO nanorods: Morphological, structural, optical, magnetic, and enhanced photocatalytic characteristics. *Ceram. Int.* **46**, 2931–2939. 10.1016/j.ceramint.2019.09.289 ‏)2020).

[58] Elsayed, I. A. & Afify, A. S. Controlling the surface morphology of ZnO nano-thin film using the spin coating technique. *Materials* **15**, 6178. 10.3390/ma15176178 (2022). 36079559 10.3390/ma15176178 PMC9458145

[59] Thi, T. U. D. *et al.* Green synthesis of ZnO nanoparticles using orange fruit peel extract for antibacterial activities. *RSC Adv.* **10**, 23899–23907. 10.1039/D0RA04926C (2020). 35517333 10.1039/d0ra04926c PMC9055061

[60] Yashni, G., Al-Gheethi, A., Mohamed, R. & Al-Sahari, M. Reusability performance of green zinc oxide nanoparticles for photocatalysis of bathroom greywater. *Water Pract. Technol.* **16**, 364–376. 10.2166/wpt.2020.118 (2021).

[61] Hunge, Y. M., Yadav, A. A., Kang, S. W., Lim, S. J. & Kim, H. Visible light activated MoS_2_/ZnO composites for photocatalytic degradation of ciprofloxacin antibiotic and hydrogen production. *J. Photochem. Photobiol. A* **434**, 114250. 10.1016/j.jphotochem.2022.114250. ‏(2023).

[62] Hunge, Y. M. *et al.* Photocatalytic degradation of bisphenol A using titanium dioxide@ nanodiamond composites under UV light illumination. *J. Colloid Interface Sci.* **582**, 1058–1066. 10.1016/j.jcis.2020.08.102 (2021). 32927171 10.1016/j.jcis.2020.08.102

[63] Hassaan, M. A., Elkatory, M. R. & El Nemr, A. Applications of photochemical oxidation in Textile Industry. *Handbook of Nanomaterials and Nanocomposites for Energy and Environmental Applications* 1–30 (2020).

[64] El Nemr, A., Helmy, E. T., Gomaa, E. A., Eldafrawy, S. & Mousa, M. Photocatalytic and biological activities of undoped and doped TiO_2_ prepared by Green method for water treatment. *J. Environ. Chem. Eng.* **7**, 103385. 10.1016/j.jece.2019.103385 (2019).

[65] Raheem, R. A., Al-gubury, H. Y., Aljeboree, A. M. & Alkaim, A. F. Photocatalytic degradation of reactive green dye by using Zinc oxide. *J. Chem. Pharm. Sci.* **9**, 1134–1138 (2016).

[66] Siddiqui, V. U. *et al.* Optimization of facile synthesized ZnO/CuO nanophotocatalyst for organic dye degradation by visible light irradiation using response surface methodology. *Catalysts* **11**, 1509. 10.3390/catal11121509 (2021).

[67] Muthukumar, C., Iype, E., Raju, K., Pulletikurthi, S. & Kumar, B. P. Sunlight assisted photocatalytic degradation using the RSM-CCD optimized sustainable photocatalyst synthesized from galvanic wastewater. *J. Mol. Struc.* **1263**, 133194. 10.1016/j.molstruc.2022.133194 (2022).

[68] Mousavi, M., Soleimani, M., Hamzehloo, M., Badiei, A. & Ghasemi, J. B. Photocatalytic degradation of different pollutants by the novel GCN-NS/black-TiO_2_ heterojunction photocatalyst under visible light: Introducing a photodegradation model and optimization by response surface methodology (RSM). *Mater. Chem. Phys.* **258**, 123912. 10.1016/J.MATCHEMPHYS.2020.123912 (2021).

[69] Galedari, M., Mehdipour, G. M. & Rashid, M. S. Photocatalytic process for the tetracycline removal under visible light: Presenting a degradation model and optimization using response surface methodology (RSM). *Chem. Eng. Res. Des.* **145**, 323–333. 10.1016/J.CHERD.2019.03.031 (2019).

[70] Hassaan, M. A., Elkatory, M. R., El-Nemr, M. A., Ragab, S. & El Nemr, A. Optimization strategy of Co_3_O_4_ nanoparticles in biomethane production from seaweeds and its potential role in direct electron transfer and reactive oxygen species formation. *Sci. Rep.* **14**, 5075. 10.1038/s41598-024-55563-y (2024). 38429365 10.1038/s41598-024-55563-y PMC11319461

[71] Hassaan, M. A. *et al.* Synthesis, characterization, optimization and application of Pisum sativum peels S and N-doping biochars in the production of biogas from Ulva lactuca. *Renew. Energy* **221**, 119747. 10.1016/j.renene.2023.119747 (2024).

[72] Ragab, S. *et al.* Experimental, predictive and RSM studies of H_2_ production using Ag-La-CaTiO_3_ for water-splitting under visible light. *Sci. Rep.* **14**, 1019. 10.1038/s41598-024-51219-z (2024). 38200036 10.1038/s41598-024-51219-z PMC10781765

[73] Hassaan, M. A. *et al.* Box-Behnken design and life cycle assessment for nickel oxide nanoparticles application in biomethane production. *Chem. Eng. J.* **474**, 145924. 10.1016/j.cej.2023.145924 (2023).

[74] Meky, A. I., Hassaan, M. A., Fetouh, H. A., Ismail, A. M. & El Nemr, A. Hydrothermal fabrication, characterization and RSM optimization of cobalt-doped zinc oxide nanoparticles for antibiotic photodegradation under visible light. *Sci. Rep.* **14**, 2016. 10.1038/s41598-024-52430-8 (2024). 38263230 10.1038/s41598-024-52430-8 PMC11231344

[75] Meky, A. I., Hassaan, M. A., Fetouh, H. A., Ismail, A. M. & El Nemr, A. Cube-shaped Cobalt-doped zinc oxide nanoparticles with increased visible-light-driven photocatalytic activity achieved by green co-precipitation synthesis. *Sci. Rep.* **13**, 19329. 10.1038/s41598-023-46464-7 (2023). 37935868 10.1038/s41598-023-46464-7 PMC10630306

[76] Hassaan, M. A. *et al.* Principles of photocatalysts and their different applications: A review. *Top. Curr. Chem.* **381**, 31. 10.1007/s41061-023-00444-7 (2023). 10.1007/s41061-023-00444-7 PMC10618379 37906318

[77] Chaker, H., Attar, A. E., Djennas, M. & Fourmentin, S. A Statistical modelingoptimization approach for efficiency photocatalytic degradation of textile azo dye using cerium-doped mesoporous ZnO: A central composite design in response surface methodology. *Chem. Eng. Res. Des.* **171**, 198–212. 10.1016/J.CHERD.2021.05.008 (2021).

[78] Aeindartehran, L. & Talesh, S. S. A. Enhanced photocatalytic degradation of Acid Blue 1 using Ni-Decorated ZnO NPs synthesized by sol-gel method: RSM optimization approach. *Ceram. Int.* **47**, 27294–27304. 10.1016/j.ceramint.2021.06.151 (2021).

[79] Eleryan, A. *et al.* Adsorption of direct blue 106 dye using zinc oxide nanoparticles prepared via green synthesis technique. *Environ. Sci. Pollut. Res.*10.1007/s11356-023-26954-x (2023). 10.1007/s11356-023-26954-x PMC10212883 37140854

[80] Hassaan, M.A., El Nemr, A. & Ragab, S. Green synthesis and application of metal and metal oxide nanoparticles. *Handbook of Nanomaterials and Nanocomposites for Energy and Environmental Applications* 1–27. doi: 10.1007/978-3-030-11155-7_125-1 (2020).

