# Supplementary material for: Central composite design and mechanism of antibiotic ciprofloxacin photodegradation under visible light by green hydrothermal synthesized cobalt-doped zinc oxide nanoparticles
Source: Sci Rep. 2024 Apr 21;14:9144. doi: 10.1038/s41598-024-58961-4 (PMC11551219; doi:10.1038/s41598-024-58961-4)
Supplement: Supplementary file 1 — Supplementary Information. [file 41598_2024_58961_MOESM1_ESM.docx]

**Supplementary material**

**Central composite design and mechanism of antibiotic ciprofloxacin photodegradation under visible light by green hydrothermal synthesized Cobalt-doped Zinc oxide nanoparticles**

Mohamed A. Hassaan^1^, Asmaa I. Meky^2,3^, Howida A. Fetouh^2^, Amel M. Ismail^2^, Ahmed El Nemr^1^*

**Table S1**. Analysis of the surface area of ZnO and Co doped ZnO.

|  | | ZnO | 5%Co-ZnO | 10%Co- ZnO | 15%Co- ZnO |
| --- | --- | --- | --- | --- | --- |
| BET | ***a*_s,BET_ (m^2^∕g)** | 6.7837 | 4.9823 | 5.3115 | 7.2564 |
|  | **Vm (cm^3^ STP)/g)** | 1.5586 | 1.1447 | 1.2203 | 1.6672 |
|  | **Mean pore diameter *P*_m_ (nm)** | 10.877 | 11.67 | 14.508 | 11.901 |
|  | **Volume of total pore *V*_T_ (cm^3^/g)** | 0.018446 | 0.014544 | 0.019265 | 0.021589 |
| BJH | ***V*_p_ (cm^3^/g)** | 0.018437 | 0.014868 | 0.019598 | 0.021593 |
|  | ***a*_p_ (m^2^/g)** | 6.8287 | 5.5432 | 5.8882 | 7.4754 |

| **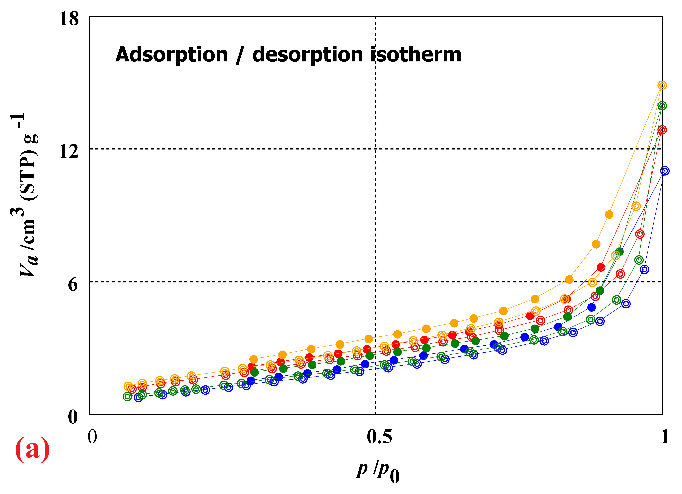** | **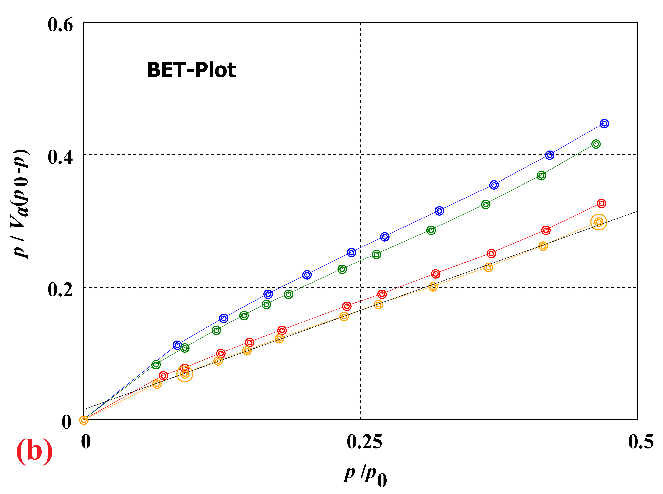** |
| --- | --- |
| **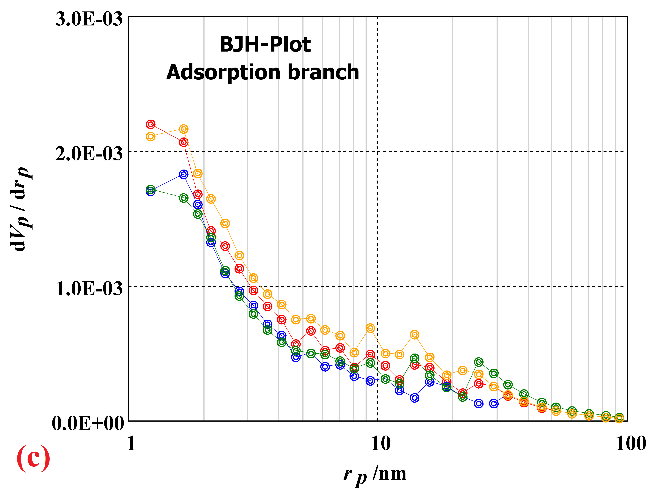** | |

**Figure S1**. (a) Adsorption–desorption; (b) BET analysis; (c) BJH analysis by adsorption of Green Hy-ZnO NPs (red), Green 5% Hy-Co-ZnO NPs (blue), Green 10% Hy-Co-ZnO NPs (green) and Green 15% Hy-Co-ZnO NPs (yellow).

**
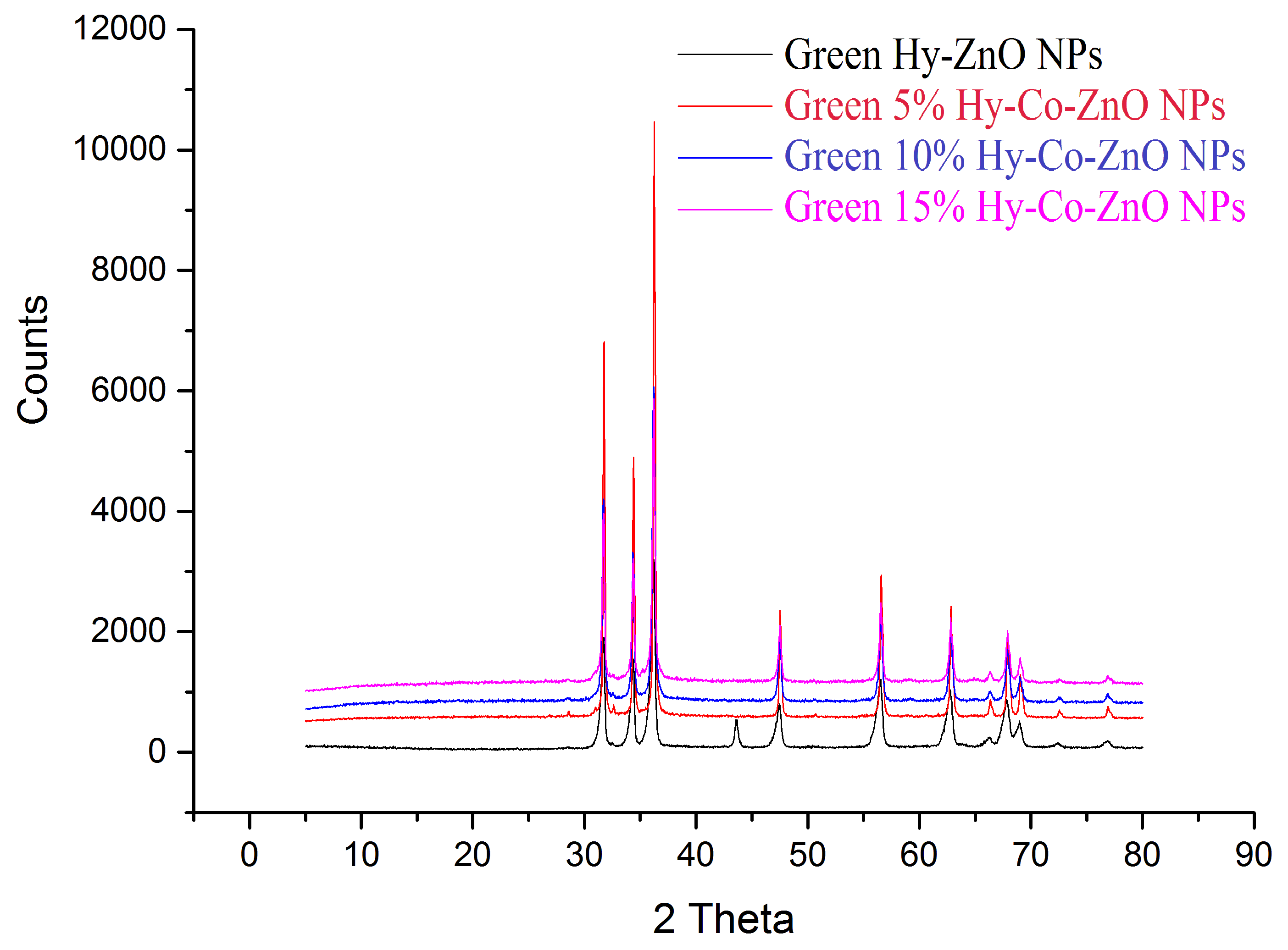
**

**Figure S2**. X-ray diffraction pattern of Green Hy-ZnO NPs (black), Green 5% Hy-Co-ZnO NPs (red), Green 10% Hy-Co-ZnO NPs (blue) and Green 15% Hy-Co-ZnO NPs (purple).

**Table S2**. Crystal size of Green Hy-ZnO and 5, 10, 15% Hy-Co-ZnO NPs (nm).

| **2Theta** | **ZnO-NPs** | **2Theta** | **5%Co-ZnO** | **2Theta** | **10%Co-ZnO** | **2Theta** | **15% Co-ZnO** |
| --- | --- | --- | --- | --- | --- | --- | --- |
| 31.328 | 43.56 | 31.759 | 78.48 | 31.743 | 77.70 | 31.736 | 75.46 |
| 33.977 | 55.98 | 34.411 | 78.24 | 34.399 | 73.85 | 34.397 | 67.54 |
| 35.801 | 45.85 | 36.244 | 71.56 | 36.227 | 67.31 | 36.223 | 66.19 |
| 47.105 | 36.12 | 47.532 | 62.49 | 47.52 | 53.56 | 47.521 | 54.62 |
| 56.164 | 39.43 | 56.575 | 59.53 | 56.563 | 60.37 | 56.561 | 57.92 |
| 62.462 | 36.18 | 62.839 | 56.70 | 62.827 | 50.84 | 62.834 | 48.87 |
| 65.974 | 43.27 | 66.373 | 77.76 | 66.347 | 64.88 | 66.335 | 65.35 |
| 67.541 | 38.48 | 68.008 | 53.25 | 67.918 | 55.49 | 67.911 | 51.42 |
| 68.673 | 39.07 | 69.061 | 55.20 | 69.058 | 57.99 | 69.052 | 52.36 |
| 72.162 | 68.18 | 72.553 | 57.45 | 72.52 | 67.35 | 72.597 | 59.28 |
| 76.572 | 43.32 | 76.897 | 71.93 | 76.906 | 55.72 | 76.966 | 49.71 |

**Table S3.** Calculated structural and optical parameters for prepared samples.

| **Sample** | **a (nm)** | **c (nm)** | **Crystallite**  **size (D) (nm)** | **disclosion density** **δ (10^-3^)** | **Microstrain** ε **(10^-3^)** | **Volume of unit cell (nm^3^)** |
| --- | --- | --- | --- | --- | --- | --- |
| **ZnO** | 0.2402 | 0.9073 | 44.49 | 0.476 | 2.337 | 0.045315 |
| **Co-ZnO 5%** | 0.2434 | 0.9115 | 65.69 | 0.195 | 1.480 | 0.046755 |
| **Co-ZnO 10%** | 0.2433 | 0.9112 | 62.28 | 0.221 | 1.574 | 0.046691 |
| **Co-ZnO 15%** | 0.2432 | 0.9120 | 58.97 | 0.228 | 1.601 | 0.46714 |

| 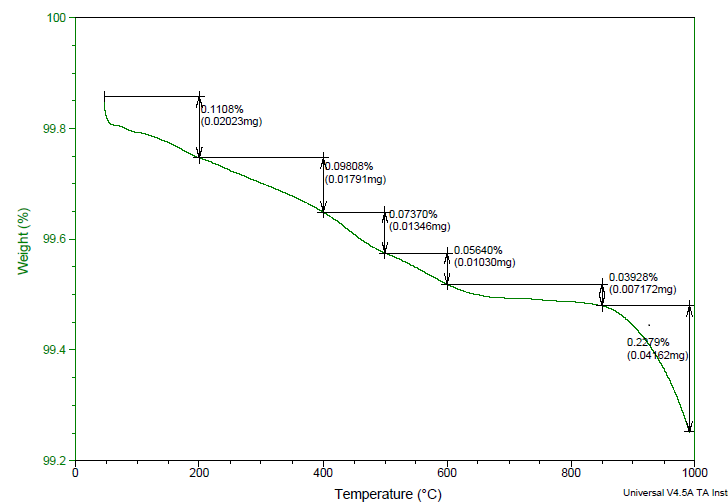 | 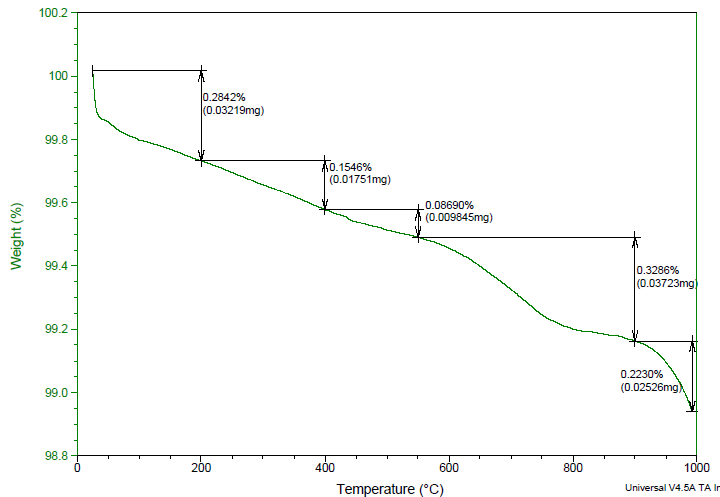 |
| --- | --- |
| (a) | (b) |
| 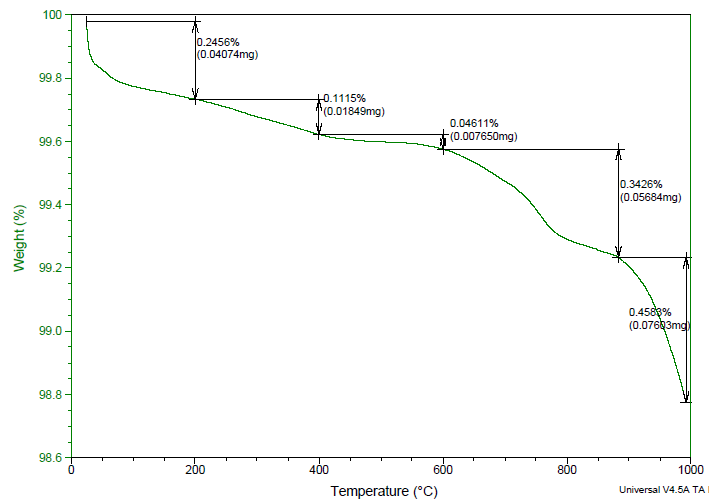 | 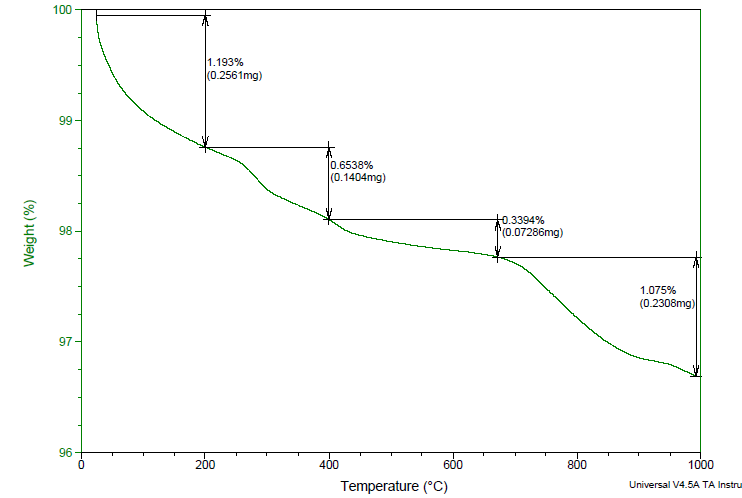 |
| (c) | (d) |

**Figure S3.** TGA analyses of **(**a) ZnO, (b) 5%Co-ZnO, (c) 10% Co-ZnO, (d) 15% Co-ZnO.

**Table S4**. Corresponding F-values and P-values and Coefficient estimate for CIPF degradation

| **Source** | **Sum of Squares** | **df** | **Mean Square** | **F-value** | **p-value** |  |
| --- | --- | --- | --- | --- | --- | --- |
| **Model** | 3937.62 | 14 | 281.26 | 5.67 | 0.0009 | significant |
| A-Catalyst dosage | 158.03 | 1 | 158.03 | 3.19 | 0.0944 |  |
| B-Antibiotic dosage | 2189.15 | 1 | 2189.15 | 44.16 | < 0.0001 |  |
| C- Shaking time | 4.53 | 1 | 4.53 | 0.0914 | 0.7666 |  |
| D-pH | 60.64 | 1 | 60.64 | 1.22 | 0.2861 |  |
| AB | 18.99 | 1 | 18.99 | 0.3830 | 0.5453 |  |
| AC | 39.92 | 1 | 39.92 | 0.8053 | 0.3837 |  |
| AD | 38.52 | 1 | 38.52 | 0.7770 | 0.3920 |  |
| BC | 48.33 | 1 | 48.33 | 0.9749 | 0.3391 |  |
| BD | 33.60 | 1 | 33.60 | 0.6779 | 0.4232 |  |
| CD | 13.78 | 1 | 13.78 | 0.2780 | 0.6057 |  |
| A² | 192.04 | 1 | 192.04 | 3.87 | 0.0678 |  |
| B² | 584.54 | 1 | 584.54 | 11.79 | 0.0037 |  |
| C² | 78.35 | 1 | 78.35 | 1.58 | 0.2279 |  |
| D² | 870.45 | 1 | 870.45 | 17.56 | 0.0008 |  |
| **Residual** | 743.55 | 15 | 49.57 |  |  |  |
| Lack of Fit | 743.55 | 10 | 74.35 |  |  |  |
| Pure Error | 0.0000 | 5 | 0.0000 |  |  |  |
| Cor Total | 4681.17 | 29 |  |  |  |  |
| **R²** | 0.8412 |  |  |  |  |  |
| **Adjusted R²** | 0.6929 |  |  |  |  |  |
| **Predicted R²** | 0.0851 |  |  |  |  |  |

| 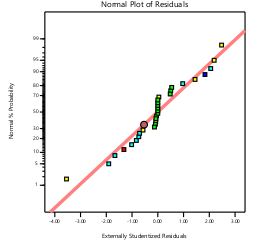 | 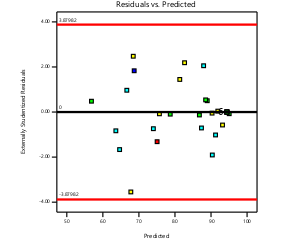 |
| --- | --- |
| **a** | **b** |
| 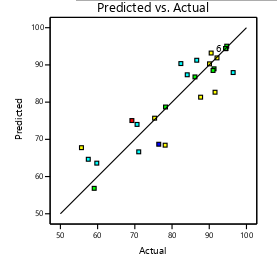 | 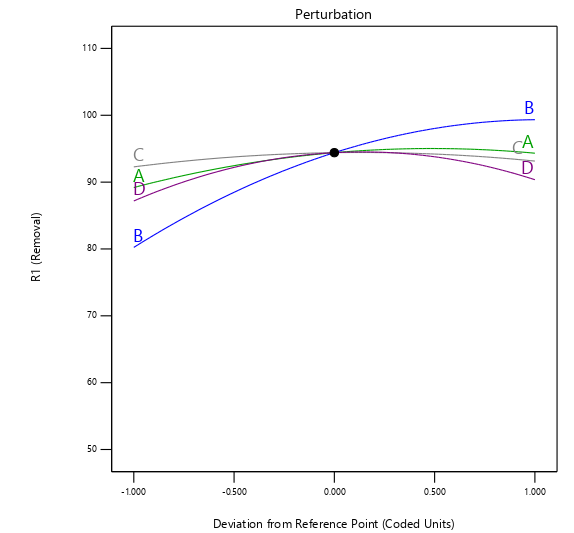 |
| **c** | **d** |

**Figure S4.** The normal probability plot (a), the graphs of residual versus predicted values (b), the graphical plot of predicted versus experimental data (c) and Perturbation plot(d).
